# Repair of topoisomerase 1–induced DNA damage by tyrosyl-DNA phosphodiesterase 2 (TDP2) is dependent on its magnesium binding

**DOI:** 10.1016/j.jbc.2023.104988

**Published:** 2023-06-29

**Authors:** Naoto Shimizu, Yusaku Hamada, Ryosuke Morozumi, Junpei Yamamoto, Shigenori Iwai, Kei-ichi Sugiyama, Hiroshi Ide, Masataka Tsuda

**Affiliations:** 1 Program of Mathematical and Life Sciences, Graduate School of Integrated Sciences for Life, Hiroshima University, Higashi-Hiroshima, Japan; 2 Program of Biomedical Science, Graduate School of Integrated Sciences for Life, Hiroshima University, Higashi-Hiroshima, Japan; 3 Graduate School of Engineering Science, Osaka University, Toyonaka, Osaka, Japan; 4 Division of Genetics and Mutagenesis, National Institute of Health Sciences, Kawasaki, Kanagawa, Japan

**Keywords:** topoisomerase 1 (TOP1), tyrosyl-DNA phosphodiesterase 1 (TDP1), tyrosyl-DNA phosphodiesterase 2 (TDP2), catalytic mechanism, divalent metal ion

## Abstract

Topoisomerases are enzymes that relax DNA supercoiling during replication and transcription. Camptothecin, a topoisomerase 1 (TOP1) inhibitor, and its analogs trap TOP1 at the 3′-end of DNA as a DNA-bound intermediate, resulting in DNA damage that can kill cells. Drugs with this mechanism of action are widely used to treat cancers. It has previously been shown that tyrosyl-DNA phosphodiesterase 1 repairs TOP1-induced DNA damage generated by camptothecin. In addition, tyrosyl-DNA phosphodiesterase 2 (TDP2) plays critical roles in repairing topoisomerase 2 (TOP2)-induced DNA damage at the 5′-end of DNA and in promoting the repair of TOP1-induced DNA damage in the absence of tyrosyl-DNA phosphodiesterase 1. However, the catalytic mechanism by which TDP2 processes TOP1-induced DNA damage has not been elucidated. In this study, we found that a similar catalytic mechanism underlies the repair of TOP1- and TOP2-induced DNA damage by TDP2, with Mg^2+^–TDP2 binding playing a role in both repair mechanisms. We show chain-terminating nucleoside analogs are incorporated into DNA at the 3′-end and abort DNA replication to kill cells. Furthermore, we found that Mg^2+^–TDP2 binding also contributes to the repair of incorporated chain-terminating nucleoside analogs. Overall, these findings reveal the role played by Mg^2+^–TDP2 binding in the repair of both 3′- and 5′-blocking DNA damage.

Topoisomerase 1 (TOP1) generates transient single-strand breaks (SSBs) in genomic DNA that relax torsional stress introduced during DNA transcription and replication (1, 2). The cleavage of DNA strands by TOP1 involves the formation of a reversible covalent intermediate between a catalytic tyrosine residue in TOP1 and the 3′-end of a SSB *via* a phosphotyrosyl bond, resulting in the TOP1 cleavage complex (TOP1cc) (3, 4). Following DNA relaxation, TOP1 is quickly removed from the DNA end upon religation of the DNA strand. Thus, the formation of TOP1ccs is transient under normal conditions. TOP1ccs are clinically important targets of anticancer drugs, which kill cancer cells by trapping the TOP1ccs. For instance, camptothecin (CPT) and its derivatives stabilize TOP1ccs by binding the TOP1–DNA interface, leading to the irreversible trapping of the TOP1cc at the 3′-end of the SSB and thereby constituting 3′-blocking lesions (5, 6, 7). When the TOP1cc persists, it has a higher probability of interfering with DNA replication and transcription machinery, leading to genomic instability (8, 9). Furthermore, defects in the repair of TOP1ccs have been directly and indirectly linked to neurological disorders (10). Thus, determining how cells repair TOP1ccs is pivotal both for exploiting anticancer therapy and understanding the etiology of related neurological diseases.

The TOP1cc is mainly removed *via* a two-step pathway that initially involves the proteasomal degradation of covalently bound TOP1 to the peptide crosslink (first step) and then followed by the removal of the residual TOP1-derived peptide from the 3′-terminus of DNA by tyrosyl-DNA phosphodiesterase 1 (TDP1) (second step). X-ray crystal structure and biochemical analyses have provided insights into the mechanism by which TDP1 removes the TOP1-derived peptide *via* 3′-tyrosyl-DNA phosphodiesterase (3′-TDP) activity without the need for divalent metal ions (11). An active site histidine of TDP1 attacks the 3′-phosphotyrosyl bond of DNA, resulting in the formation of a covalent bond between the histidine and the 3′-phosphate as well as the concomitant release of the TOP1-derived peptide from DNA (12, 13). Subsequently, another active site histidine of TDP1 activates a water molecule that attacks the 3′-phosphate of DNA to break the covalent bond between the histidine of TDP1 and the 3′-phosphate of DNA, thereby releasing the DNA substrate from TDP1 (14). In addition to the 3′-phosphotyrosyl bond, TDP1 also hydrolyzes various 3′-blocking lesions, which are produced by chain-terminating nucleoside analogs (CTNAs) (15, 16, 17, 18). Thus, TDP1 has a broad range of DNA repair activities and is a potential drug target in anticancer therapy (19).

Topoisomerase 2 (TOP2) resolves DNA torsional stress *via* a cleavage–religation mechanism in which TOP2 induces transient DNA double-strand breaks (DSBs) (20). This transient cleavage links TOP2 to the 5′-end of DNA, forming a TOP2–DNA covalent intermediate commonly called a TOP2 cleavage complex (TOP2cc) (21). Etoposide (ETP), a frontline anticancer drug, “freezes” the reaction intermediate, irreversibly trapping the TOP2cc (20). Tyrosyl-DNA phosphodiesterase 2 (TDP2) possesses robust 5′-tyrosyl-DNA phosphodiesterase (5′-TDP) activity, which plays a critical role in the repair of TOP2ccs (22), as demonstrated in a study showing that TDP2 contributes to ETP resistance (23). Similar to the aforementioned two-step TOP1cc repair mechanism, TOP2cc repair mainly progresses *via* a pathway involving the initial proteasomal degradation of the TOP2cc to the crosslinked peptide (Fig. S1*A*), which are then removed by TDP2 (24). Biochemical analyses have revealed that TDP2 differs from TDP1 in that the binding of a divalent Mg^2+^ to TDP2 is required for 5′-TDP activity to progress (22, 25, 26). X-ray structure studies have shown that Mg^2+^ is stabilized by Glu152 of TDP2 (the amino acid numbering is that of the human protein) and is optimally positioned to interact with the 5′-phosphate and form a pretransition state intermediate (Fig. S1*B*) (27, 28, 29). Nucleophilic water, which is activated by Asp262 of TDP2, attacks the 5′-phosphate of the tyrosyl-DNA adduct (28, 29). As the reaction proceeds, the O-P bond between the tyrosine and the phosphate lengthens and ultimately breaks, forming a free peptide with a tyrosine end and DNA with a 5′-phosphate end (Fig. S1*C*) (11). Biochemical analyses have also revealed that a mutation at residue Glu152 gives rise to a catalytically inactive protein in terms of 5′-TDP activity (22, 25, 28). The mutation affecting Mg^2+^ binding impairs catalysis on the 5′-phosphotyrosyl-DNA substrates *in vitro*; however, it is unclear whether the genomic mutation at residue Glu152 affects the repair of TOP2-mediated DNA damage *in vivo*.

TDP2 also possesses weak 3′-tyrosyl-DNA phosphodiesterase activity; thus, it plays an important role in the repair of TOP1ccs in the absence of TDP1 (23, 30). We previously found that TDP2 promotes the second but not the first step of the aforementioned TOP1cc repair pathway in the absence of TDP1 (30). Regarding human TK6 cells, we also found that *TDP1*^*−/−*^*/TDP2*^*−/−*^ cells were more sensitive than *TDP1*^*−/−*^ or *TDP2*^*−/−*^ cells to various CTNAs, suggesting that TDP2 plays a novel role in the removal of CTNAs incorporated at the 3′-ends of DNA (30). However, the mechanism by which TDP2 processes the 3′-phosphotyrosyl bond and various types of 3′-blocking lesions remains unclear.

In the present study, we aimed to elucidate the 3′-TDP activity of human TDP2 both *in vitro* and *in vivo*. We found that the E152Q, D262N, W297A, R206A, and D350N mutations of the purified recombinant TDP2 protein abolish both the 5′- and 3′-TDP activities. Furthermore, we revealed that the genomic E152Q mutation of the *TDP2* gene impairs the second step of the TOP1cc repair pathway in TDP1-deficient human TK6 cells. The E152Q mutation also renders TDP1-deficient TK6 cells sensitive to CPT and various agents that produce 3′-blocking lesions. Taken together, our findings indicate that the binding of Mg^2+^ to TDP2 plays a critical role in the repair of both 5′- and 3′-blocking lesions.

## Results

### The 3′- and 5′-TDP activities of TDP2 have common divalent metal ion requirements

**Figure 1:**
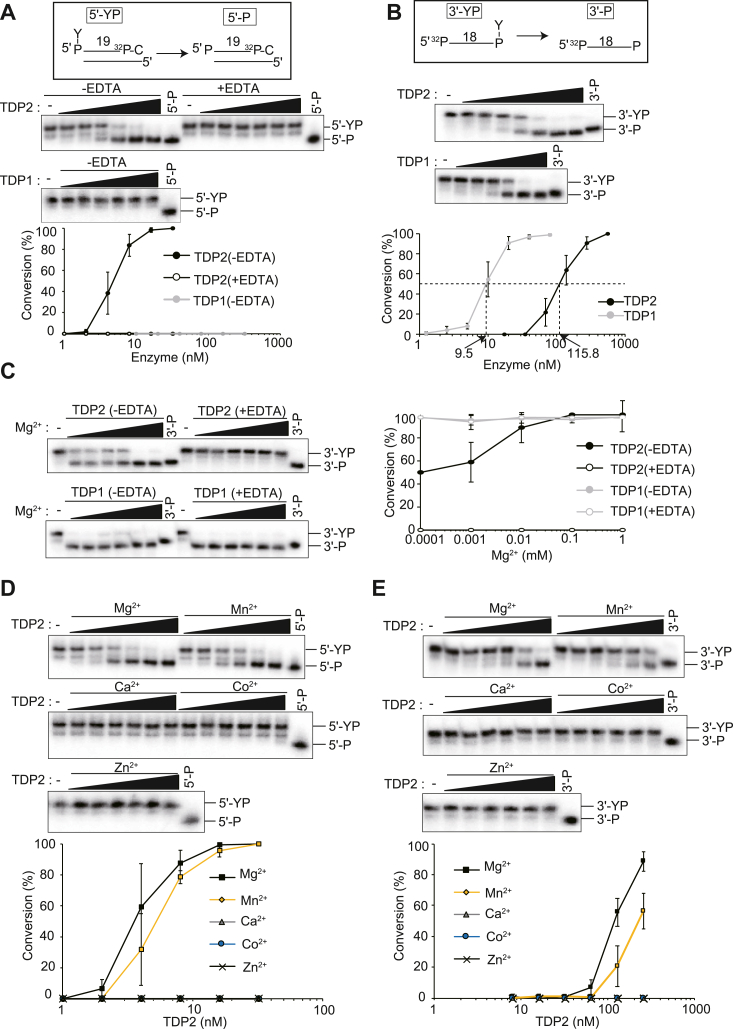
**Common divalent metal ion requirements of the 3′- and 5′-TDP activities of TDP2.***A*, 5′-TDP activity of TDP2. *Top*: Schematic representation of the 5′-TDP activity assay. 5′-TDP activity catalyzes the hydrolysis of the 5′-phosphotyrosyl bond and converts the duplex 5′-YP substrate (19-mer) to a product with a 5′-phosphate end (5′-P; 19-mer). *Middle*: Representative gels showing the results of 5′-TDP activity assays. The duplex 5′-YP substrate was incubated with TDP2 (0, 1, 2, 4, 8, 16, and 32 nM) or TDP1 (0, 10, 20, 40, 80, 160, and 320 nM) in a TDP reaction buffer containing 1 mM MgCl_2_ in the absence (−) or presence (+) of 50 mM EDTA at 37 °C for 10 min. Products were separated *via* denaturing PAGE and detected using autoradiography. Positions of the ^32^P-radiolabeled substrate (5′-YP) and the product (5′-P) are indicated. A standard marker for 5′-P (separately prepared) was also separated in the rightmost lanes of the gels. *Bottom*: Conversion (%) of the 5′-YP substrate to the product (5′-P) at different TDP1 and TDP2 concentrations in the absence (−) or presence (+) of EDTA. *B*, 3′-TDP activity of TDP2. *Top*: Schematic representation of the 3′-TDP activity assay. 3′-TDP activity catalyzes the hydrolysis of the 3′-phosphotyrosyl bond and converts the single-stranded 3′-YP substrate (18-mer) to a product with a 3′-phosphate end (3′-P; 18-mer). *Middle*: Representative gels showing the results of 3′-TDP activity assays. The single-stranded 3′-YP substrate was incubated with TDP1 (0, 1.25, 2.5, 5, 10, 20, 40, and 80 nM) or TDP2 (0, 17.3, 34.6, 69.2, 138.4, 276.8, and 553.6 nM) in a TDP reaction buffer containing 1 mM MgCl_2_ at 37 °C for 10 min. Products were separated *via* denaturing PAGE. Positions of the ^32^P-radiolabeled substrate (3′-YP) and the product (3′-P) are indicated. A standard marker for 3′-P (separately prepared) was also separated in the rightmost lanes of the gels. *Bottom*: Conversion (%) of the 3′-YP substrate to the product (3′-P) at different TDP1 and TDP2 concentrations. The broken line indicates 50% conversion. *C*, effect of Mg^2+^ concentration on the 3′-TDP activity of TDP2. *Left*: The 3′-YP substrate was incubated with 553.6 nM TDP2 (*upper*) or 80 nM TDP1 (*lower*) in a TDP reaction buffer containing MgCl_2_ (0.0001, 0.001, 0.01, 0.1, and 1 mM) in the absence (−) or presence (+) of 50 mM EDTA, and the products were separated *via* denaturing PAGE. The rightmost lane of the gels indicates the standard marker for the product (3′-P). *Right*: Conversion (%) of the 3′-YP substrate to the product (3′-P) by TDP2 and TDP1 at different concentrations of Mg^2+^ in the absence (−) or presence (+) of EDTA. *D*, effects of different divalent metal ions on the 5′-TDP activity of TDP2. *Upper*: Duplex 5′-YP substrate was incubated with TDP2 (0, 1, 2, 4, 8, 16, and 32 nM) in a TDP reaction buffer containing 1 mM Mg^2+^, Mn^2+^, Ca^2+^, Co^2+^, or Zn^2+^, and the products were separated *via* denaturing PAGE. *Lower*: Conversion (%) of the 5′-YP substrate to the product (5′-P) by TDP2 in the presence of Mg^2+^, Mn^2+^, Ca^2+^, Co^2+^, or Zn^2+^. *E*, effects of different divalent metal ions on the 3′-TDP activity of TDP2. *Upper*: Single-stranded 3′-YP substrate was incubated with TDP2 (8, 16, 32, 64, 129, and 257 nM) in a TDP reaction buffer containing 1 mM Mg^2+^, Mn^2+^, Ca^2+^, Co^2+^, or Zn^2+^, and the products were separated *via* denaturing PAGE. *Lower*: Conversion (%) of the 3′-YP substrate to the product (3′-P) by TDP2 in the presence of Mg^2+^, Mn^2+^, Ca^2+^, Co^2+^, or Zn^2+^. The data in panels *A*–*E* are the means ± SDs of at least three independent experiments. TDP, tyrosyl-DNA phosphodiesterase.

To investigate the catalytic mechanism of TDP2 *in vitro*, we used a recombinant human TDP2 protein purified from *Escherichia coli* (Fig. S2*A*). First, we confirmed the intrinsic 5′-TDP activity of TDP2 using a duplex DNA substrate (5′-YP) bearing a 5′-phosphotyrosine residue at the DSB end (Fig. 1*A*, top). The duplex 5′-YP was incubated with TDP2 (or recombinant TDP1 protein for comparison), and the products were analyzed using denaturing PAGE (Fig. 1*A*, middle). TDP2 hydrolyzed the 5′-phosphotyrosyl bond of 5′-YP and converted 5′-YP to a product with a 5′-phosphate end (5′-P), thereby demonstrating intrinsic 5′-TDP activity. The 5′-TDP activity of TDP2 was evident in the absence of EDTA but absent in the presence of EDTA (Fig. 1*A*, middle and bottom). Conversely, TDP1 failed to hydrolyze the 5′-phosphotyrosyl bond of 5′-YP.

Next, we analyzed the 3′-TDP activity of TDP2. A single-stranded DNA substrate (3′-YP) bearing a phosphotyrosine residue at the 3′-end (3′-pTyr) was used as a substrate (Fig. 1*B*, top). The 3′-YP substrate was incubated with TDP2 (or TDP1 for comparison), and the products were analyzed using denaturing PAGE. TDP1, used as the control, hydrolyzed the 3′-phosphotyrosyl bond of 3′-YP and converted 3′-YP to a product with a 3′-phosphate end (3′-P), demonstrating the intrinsic 3′-TDP activity of TDP1 (Fig. 1*B*, middle). Consistent with previous findings (22), TDP2 also hydrolyzed the 3′-phosphotyrosyl bond of 3′-YP, indicating 3′-TDP activity. The 50% conversion of the 3′-YP substrate was obtained at 9.5 nM TDP1 and 115.8 nM TDP2 (Fig. 1*B*, bottom); thus, the 3′-TDP activity of TDP2 was 12-fold less efficient than that of TDP1.

We further analyzed the 3′-TDP activity of TDP2 by varying the concentration of Mg^2+^ (0.0001–1 mM MgCl_2_) in the reaction buffer (Fig. 1*C*). The 3′-TDP activity of TDP2 increased when the concentration of Mg^2+^ was increased but was abolished by the addition of 50 mM EDTA. Conversely, the 3′-TDP activity of TDP1 was independent of the Mg^2+^ concentration and the presence or absence of EDTA, indicating that the 3′-TDP activity of TDP1 did not require divalent cations.

To determine the effects of different divalent cations on the 5′- and 3′-TDP activities of TDP2, we incubated duplex 5′-YP and single-stranded 3′-YP substrates with TDP2 in the presence of various divalent cations (*i.e.*, Mg^2+^, Mn^2+^, Co^2+^, Ca^2+^, and Zn^2+^) and analyzed the products. TDP2 exhibited 5′-TDP and 3′-TDP activity in the presence of Mg^2+^ or Mn^2+^ ions but not in the presence of Co^2+^, Ca^2+^, or Zn^2+^ ions (Fig. 1, *D* and *E*). Therefore, the 3′- and 5′-TDP activities of TDP2 have common metal ion requirements.

TOP1-induced DNA damage can occur during replication or transcription (4). TOP1cc can be converted into various DNA products bearing a 3′-pTyr such as nicked duplex DNA and other related products (ssDNA, blunt-end DNA, and 5′-overhang DNA; Fig. S3*A*). To better understand the activity of TDP2 for various 3′-pTyr substrates, we prepared ssDNA, blunt-end DNA, 5′-overhang DNA, and nicked duplex DNA bearing 3′-pTyr. These substrates exhibited different mobilities in native PAGE (Fig. S3*B*). We then explored the ability of TDP2 (and TDP1 for comparison) to remove the tyrosine residue at 3′-pTyr. As shown in the previous paper (31), the 3′-TDP activity of TDP1 for nicked duplex DNA was lower than those for ssDNA, blunt-end DNA, and 5′-overhang DNA (Fig. S3*C*). Importantly, the 3′-TDP activity of TDP2 for nicked duplex DNA was also low relative to those for ssDNA, blunt-end DNA, and 5′-overhang DNA (Fig. S3*D*), indicating that both TDP1 and TDP2 have a similar substrate specificity.

### Mg^2+^–TDP2 binding is required for 5′-TDP activity

**Figure 2:**
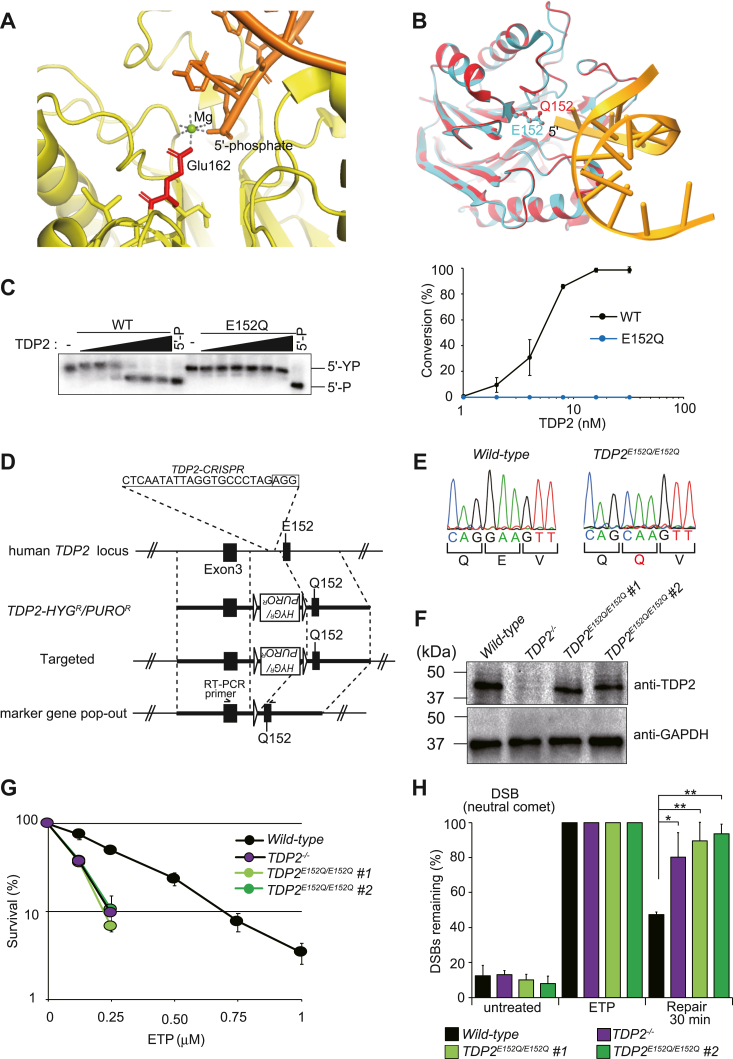
**Role of Mg**^**2+**^**–TDP2 binding in 5**′**-TDP activity.***A*, structure of mouse TDP2 in complex with Mg^2+^ and 5′-phosphate DNA. The crystal structure of the TDP2 complex (PDB#4GZ1) was obtained from Protein Data Bank (PDB). DNA is shown in *orange* with the indicated 5′-end, Mg^2+^ is shown as a *green sphere*, the carboxylate group of Glu162 (corresponding to Glu152 in human) is marked in *red*, and hydrogen bonds or metal-coordination interactions are denoted by *dotted lines*. *B*, structural comparison of the WT and E152Q mutant forms of human TDP2. A computational structural model of the E152Q mutant was obtained *via* homology modeling (SWISS-MODEL) based on the crystal structure of WT human TDP2 (PDB: 5INO). Superpositions of the WT (*cyan*) and E152Q mutant (*red*) are shown. E152 in the WT and Q152 in the mutant are shown as *ball sticks*. DNA is shown in *orange* with the indicated 5′-end. *C*, E152Q mutation of TDP2 abolishes 5′-TDP activity. *Left*: WT or E152Q TDP2 (0, 1, 2, 4, 8, 16, and 32 nM) were incubated with the duplex 5′-YP substrate in a TDP reaction buffer containing 1 mM Mg^2+^ at 37 °C for 10 min, and the products were analyzed using denaturing PAGE. *Right*: Conversion (%) of the 5′-YP substrate to the product (5′-P) by WT or E152Q TDP2. Data are means ± SDs of three independent experiments. *D*, schematic representation of the *TDP2* locus in TK6 cells and the structure of the gene-targeting constructs. Genomic region around exons 3 and 4 in the WT allele is shown in the *top* line. *Closed solid boxes* indicate the coding regions of exons 3 and 4. E152 is encoded in exon 4 of the human *TDP2* gene. TDP2-CRISPR vector expresses gRNA targeting the intron between exons 3 and 4. TDP2 targeting vector (*TDP2-HYG*^*R*^*/PURO*^*R*^) is shown in the second line. *Bold lines* indicate homology arms. To create a knock-in allele, a sequence change from GAA (E152) to CAA (Q152) was incorporated into the *right* arm of the targeting vector. *HYG*^*R*^ and *PURO*^*R*^ designate the hygromycin- and puromycin-resistant gene expression cassettes, respectively. *HYG*^*R*^ and *PURO*^*R*^ are flanked by lox^P^ sites (*open triangles*). Third line shows the correctly targeted allele; fourth line indicates the allele after “popping out” of the marker gene cassette *via* the transfection of Cre recombinase. *Arrows* indicate the primers used for RT-PCR. *E*, sequence chromatograms of the RT-PCR products amplified from WT or E152Q mutant mRNA. Codon for E152 (GAA) was changed to that for Q152 (CAA). *F*, analysis of the TDP2 protein level in WT, *TDP2*^*−/−*^, and *TDP2*^*E152Q/E152Q*^ cells. Whole extracts were prepared from WT, *TDP2*^*−/−*^, and *TDP2*^*E152Q/E152Q*^ (clones #1 and #2) cells and separated using SDS-PAGE. TDP2 protein was detected using anti-TDP2 antibody. GAPDH was used as the loading control. *G*, survival curves of WT, *TDP2*^*−/−*^, and *TDP2*^*E152Q/E152Q*^ TK6 cells after treatment with ETP. Cells were treated with the indicated concentrations of ETP for 3 h. Cell survival was measured using colony forming assays as described in the Experimental procedures*.* Survival of untreated cells was set as 100%. Data are means ± SDs of three independent assays. *H*, repair of TOP2cc-induced DSBs in ETP-treated cells. Cells were incubated with 1 μM ETP for 60 min at 37 °C and then in ETP-free media for 30 min to allow DSB repair. DSBs were quantified using neutral comet assays in which tail moments were measured for 50 cells/sample/experiment. Median tail moments were standardized to those at 0 min of repair (bars on ETP). Percentages of the remaining DSBs after 30 min were calculated relative to those at 0 min and are presented as fractions of the DSBs remaining. Data are means ± SDs from three biological replicates. Significant differences were identified using Student’s *t* test: ∗*p* < 0.05 and ∗∗*p* < 0.01. Typical neutral comet images and the raw data of tail moments before standardization are shown in Fig. S5, *A* and *B*, respectively. DSB, double-strand break; ETP, etoposide; TDP, tyrosyl-DNA phosphodiesterase; TOP, topoisomerase; TOP2cc, TOP2 cleavage complex.

In mouse TDP2, the coordination between the carboxylate group of Glu162 (corresponding to Glu152 in human TDP2) and Mg^2+^ is critical for forming a transition state intermediate with 5′-phosphate to express 5′-TDP activity (Fig. 2*A*) (28). Thus, Glu152 may also be critical for the 5′- and 3′-TDP activities of human TDP2. Substituting Gln152 for Glu152 (*i.e.*, the E152Q mutation) to eliminate the carboxylate group of Glu152 would provide a system for studying the catalytic mechanisms of TDP2; however, altering an amino acid sequence can change the folding and stability of a protein (32, 33). Thus, we first determined whether the E152Q mutation affects the protein folding of TDP2 using homology modeling of the protein structure *via* SWISS-MODEL. The predicted overall structure of the TDP2 E152Q mutant was almost identical to that of the WT TDP2 (Fig. 2*B*), suggesting that the substitution had little effect on protein folding. Accordingly, we purified the recombinant protein of the TDP2 E152Q mutant from *E. coli* (Fig. S2*B*). The WT and E152Q mutant TDP2 proteins were then incubated with the duplex 5′-YP substrate, and the products were analyzed using denaturing PAGE. We found that the E152Q mutation completely abolished the 5′-TDP activity of TDP2 (Fig. 2*C*).

To investigate the role of Mg^2+^–TDP2 binding *in vivo*, we inserted a point mutation (E152Q) into the TDP2 allelic gene in human TK6 cells using the CRISPR/Cas9 system (Figs. 2*D* and S4*A*). We verified the successful insertion of the mutations using RT-PCR and nucleotide sequencing (Fig. 2*E*). The resulting *TDP2*^*E152Q/E152Q*^ cells (two clones: #1 and #2) exhibited normal TDP2 protein levels (Fig. 2*F*) and proliferated with normal kinetics (Fig. S4*B*). However, *TDP2*^*E152Q/E152Q*^ cells were hypersensitive to ETP, and the ETP sensitivity of *TDP2*^*E152Q/E152Q*^ cells was comparable to that of *TDP2*^*−/−*^ cells (Fig. 2*G*). We also assessed DSB repair kinetics in WT and mutant TK6 cells. Specifically, WT, *TDP2*^*−/−*^, and *TDP2*^*E152Q/E152Q*^ cells were pulse-exposed to ETP, generating TOP2cc-induced DSBs, after which the cells were allowed to recover in a drug-free medium. DSBs were analyzed using neutral comet assays. Compared with the WT cells, the *TDP2*^*E152Q/E152Q*^ and *TDP2*^*−/−*^ cells exhibited a marked delay in the repair of DSBs (Figs. 2*H* and S5). Thus, *TDP2*^*E152Q/E152Q*^ cells apparently exhibit a defect in the repair of TOP2-induced DSBs. Collectively, these findings indicate that the binding of Mg^2+^ to TDP2 is required for 5′-TDP activity *in vitro* and *in vivo*.

### *TDP2* E152Q mutation abolishes 3′-TDP activity and impairs the second step of the TOP1cc repair pathway in the absence of TDP1

**Figure 3:**
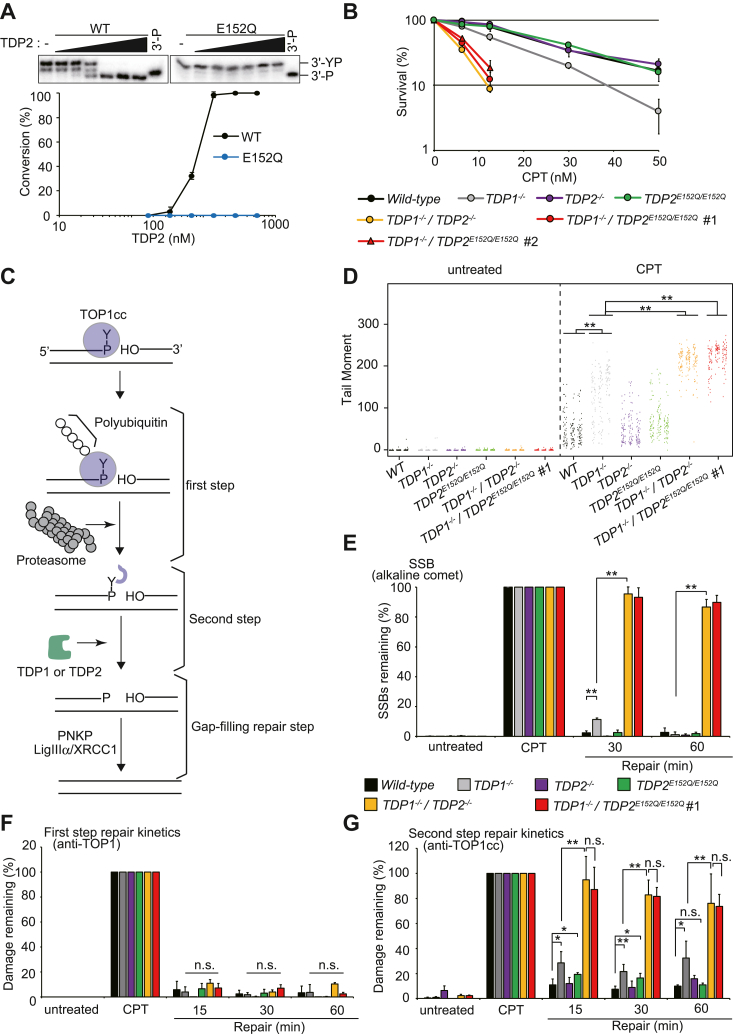
**E152Q mutation that ablates Mg**^**2+**^**–TDP2 binding also abolishes 3′-TDP activity *in vitro* and *in vivo*.***A*, E152Q mutation abolishes the 3′-TDP activity of the purified TDP2 protein. WT or E152Q TDP2 (0, 17.3, 34.6, 69.2, 138.4, 276.8, and 553.6 nM) were incubated with the 3′-YP substrate in a TDP reaction buffer containing 1 mM Mg^2+^ at 37 °C for 10 min, and the products were analyzed using denaturing PAGE (*upper*). Conversion (%) of 3′-YP to the product (3′-P) is plotted against the TDP2 concentration (*lower*). *B*, survival curves of WT and *TDP1/TDP2*-mutated TK6 cells following CPT treatment. Survival rates of WT, *TDP1*^*−/−*^, *TDP2*^*−/−*^, *TDP2*^*E152Q/E152Q*^, *TDP1*^*−/−*^*/TDP2*^*−/−*^, and *TDP1*^*−/−*^*/TDP2*^*E152Q/E152Q*^ (clones #1 and #2) cells were measured using colony-forming assays (as described for ETP treatment in Fig. 2*G*). *C*, model of TOP1cc repair. In the first step, the irreversibly trapped TOP1cc is polyubiquitinated and proteolyzed to the peptide by the proteasome. In the second step, the phosphotyrosyl bond linking DNA and the TOP1-derived peptide is hydrolyzed by TDP1 or TDP2, resulting in a 3′-phosphate end. Polynucleotide kinase/phosphatase (PNKP) removes the 3′-phosphate to generate a 3′-OH end and phosphorylates the 5′-OH end to produce a 5′-phosphate. 3′-OH and 5′-phosphate ends are ligated by LIGIIIα in the presence of XRCC1, that is, the gap-filling repair step. *D*, tail moments (raw data) of CPT-treated and untreated cells in alkaline comet assays. WT, *TDP1*^*−/−*^, *TDP2*^*−/−*^, *TDP2*^*E152Q/E152Q*^, *TDP1*^*−/−*^*/TDP2*^*−/−*^, and *TDP1*^*−/−*^*/TDP2*^*E152Q/E152Q*^ (#1) cells were treated with 25 μM CPT for 60 min or left untreated, and the tail moments were measured without a postincubation step using alkaline comet assays. In total, 50 cells were analyzed for each sample, and experiments were conducted in triplicate for each cell type. Tail moments of individual cells (an arbitrary number of SSBs) of each cell type from three experiments are plotted vertically in three separate columns. Significant differences were determined using a Wilcoxon rank sum test: ∗∗*p* < 0.01. *E*, repair kinetics of SSBs in WT and *TDP1/TDP2*-mutated TK6 cells. Indicated cells were exposed to 25 μM CPT for 60 min and then incubated in CPT-free culture media for 30 or 60 min. Tail moments were measured using alkaline comet assays (as in *panel D*). Tail moments were standardized to those at 0 min of repair (bars on CPT). Percentages of remaining SSBs after 30 and 60 min were calculated relative to those at 0 min and are presented as the fractions of SSBs remaining. Data are means ± SDs from three biological replicates. Significant differences were identified using Student’s *t* test: ∗∗*p* < 0.01. Typical alkaline comet images and the raw data of tail moments before standardization are shown in Fig. S7, *A* and *B*, respectively. *F* and *G*, repair kinetics of CPT-induced TOP1ccs in WT and *TDP1/TDP2*-mutated TK6 cells. Cells were treated with CPT (as in *panel D*), and genomic DNA was isolated after the indicated postrepair incubation times. DNA was slot-blotted on a nitrocellulose membrane, and the membrane was probed with anti-TOP1 (*F*) or anti-TOP1cc (*G*) antibodies. Quantities of TOP1 (*F*) and TOP1-derived peptides (*G*) covalently linked to DNA were standardized to those after 0 min of repair. Remaining damage is presented for each cell type (as in *panel E*; also see panel *E* for the bar colors). Significant differences were identified using Student’s *t* test: ∗∗*p* < 0.01, ∗*p* < 0.05, and n.s. = not significant. CPT, camptothecin; ETP, etoposide; SSB, single-strand break; TDP, tyrosyl-DNA phosphodiesterase; TOP, topoisomerase; TOP1cc, TOP1 cleavage complex.

To determine the role played by Glu152 of TDP2 in TOP1cc repair, we analyzed the 3′-TDP activity of the purified E152Q TDP2 mutant protein. The TDP2 WT and E152Q mutant were incubated with the 3′-YP substrate, and the products were analyzed using denaturing PAGE. The E152Q mutation abolished the hydrolase activity required for the 3′-phosphotyrosyl bond of 3′-YP, confirming the essential role of E152 in 3′-TDP activity (Fig. 3*A*).

In cells, TDP2 contributes to TOP1cc repair in the absence of TDP1 (23, 30). To determine the role played by Glu152 of TDP2 in TOP1cc repair *in vivo*, we generated *TDP1*^*−/−*^*/TDP2*^*E152Q/E152Q*^ TK6 cells and compared the CPT and ETP sensitivities of WT, *TDP1*^*−/−*^, *TDP2*^*−/−*^, *TDP2*^*E152Q/E152Q*^, *TDP1*^*−/−*^*/TDP2*^*−/−*^, and *TDP1*^*−/−*^*/TDP2*^*E152Q/E152Q*^ cells. *TDP1*^*−/−*^ cells but not *TDP2*^*−/−*^ and *TDP2*^*E152Q/E152Q*^ cells were sensitive to CPT (Fig. 3*B*). Moreover, the CPT sensitivity of *TDP1*^*−/−*^*/TDP2*^*−/−*^ cells was higher than that of *TDP1*^*−/−*^ cells. Remarkably, *TDP1*^*−/−*^*/TDP2*^*E152Q/E152Q*^ cells and *TDP1*^*−/−*^*/TDP2*^*−/−*^ cells exhibited the same sensitivity to CPT. Taken together, these results suggest that Glu152 of TDP2 contributes to TOP1cc repair in the absence of TDP1. Conversely, *TDP2*^*−/−*^ and *TDP2*^*E152Q/E152Q*^ cells were sensitive to ETP (Fig. S6). However, the addition of the TDP1 mutation (*TDP1*^*−/−*^) to *TDP2*^*−/−*^ and *TDP2*^*E152Q/E152Q*^ cells did not increase their sensitivity to ETP (Fig. S6). Thus, Glu152 of TDP2 participates in the repair of the TOP2cc *in vivo*, and TDP1 plays no role in this repair process.

Trapped TOP1cc is eliminated from DNA *via* a two-step pathway that is followed by a gap-filling repair step (Fig. 3*C*). The kinetics of gap-filling repair (*i.e.*, SSB repair) can be monitored using alkaline comet assays (34). To investigate the gap-filling repair reaction in TK6 cells, we pulse-exposed WT, *TDP1*^*−/−*^, *TDP2*^*−/−*^, *TDP2*^*E152Q/E152Q*^, *TDP1*^*−/−*^*/TDP2*^*−/−*^, and *TDP1*^*−/−*^*/TDP2*^*E152Q/E152Q*^ cells to CPT and then analyzed SSBs. As previously reported for *TDP1*-depleted mouse cells (35), a higher number of SSBs accumulated in *TDP1*^*−/−*^ cells than in WT cells immediately after CPT treatment (Figs. 3*D* and S7). Additionally, more SSBs accumulated in *TDP1*^*−/−*^*/TDP2*^*−/−*^ cells than in *TDP1*^*−/−*^ or *TDP2*^*−/−*^ cells. Notably, although *TDP2*^*E152Q/E152Q*^ cells did not accumulate SSBs, more SSBs accumulated in *TDP1*^*−/−*^*/TDP2*^*E152Q/E152Q*^ cells than in *TDP1*^*−/−*^ cells (Fig. 3*D*). We also analyzed the time course of gap-filling repair, quantifying the SSBs in cells 30 and 60 min after CPT treatment. The *TDP2* E152Q mutation alone did not lead to significant defects in gap-filling repair kinetics (Fig. 3*E*); however, *TDP1*^*−/−*^*/TDP2*^*E152Q/E152Q*^ cells exhibited a significant delay in the gap-filling repair process compared with that of *TDP1*^*−/−*^ or *TDP2*^*E152Q/E152Q*^ cells (Fig. 3*E*). Hence, Glu152 of TDP2 plays a pivotal role in the repair of TOP1cc-associated SSBs in the absence of TDP1.

To separately investigate the kinetics of the first and second steps of the TOP1cc repair pathway (Fig. 3*C*), we previously developed an assay system in which monoclonal antibodies against the TOP1 protein (anti-TOP1) and the TOP1-catalytic site peptide–DNA complex (anti-TOP1cc) are employed, respectively (30). Using this assay system, we revealed that TDP2 promotes the second but not the first step of the TOP1cc repair pathway in the absence of TDP1 *in vivo* (30). Thus, we hypothesized that the E152Q mutation of TDP2 would also impair the second step of the TOP1cc repair process in the absence of TDP1. The levels of TOP1 protein and TOP1-catalytic site peptide covalently attached to DNA were quantified using anti-TOP1 and anti-TOP1cc, respectively, 15 to 60 min after CPT treatment. The repair kinetics of the first step were similar in WT, *TDP1*^*−/−*^, *TDP2*^*−/−*^, *TDP2*^*E152Q/E152Q*^, *TDP1*^*−/−*^*/TDP2*^*−/−*^, and *TDP1*^*−/−*^*/TDP2*^*E152Q/E152Q*^ cells (Figs. 3*F* and S8*A*). However, a delay in the kinetics of the second step was observed in *TDP1*^*−/−*^*/TDP2*^*−/−*^ cells, and an equivalent delay was observed in *TDP1*^*−/−*^*/TDP2*^*E152Q/E152Q*^ cells (Figs. 3*G* and S8*B*). These results indicate that Glu152 of TDP2 is required *in vivo* for the hydrolysis of the phosphotyrosyl bond between DNA and TOP1-derived peptides following the proteasomal degradation of the TOP1cc.

### Catalytic mechanism underlying the 3′-TDP activity of TDP2

**Figure 4:**
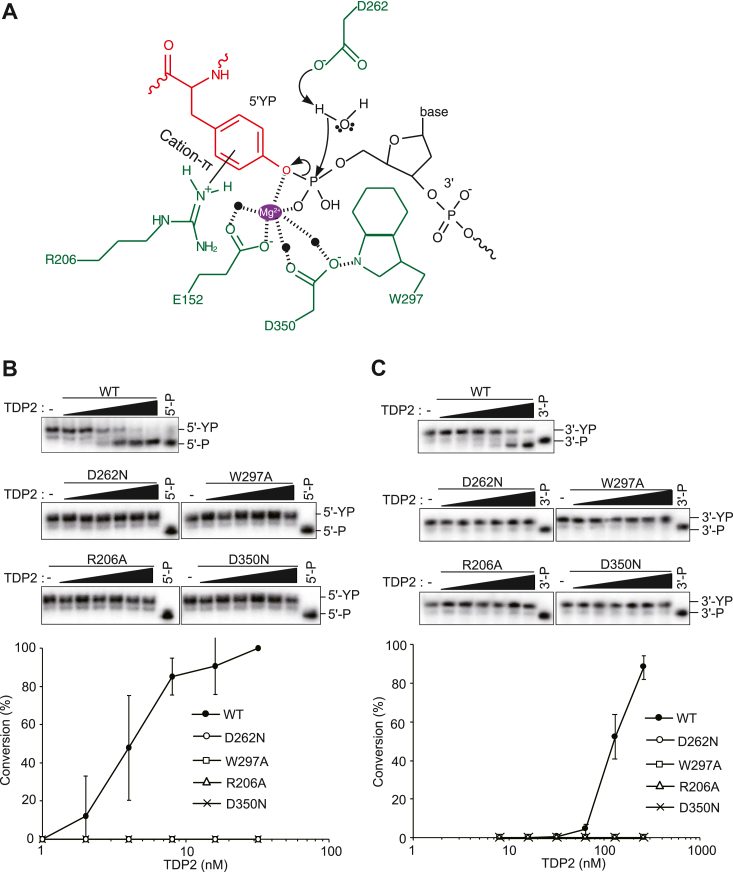
**Putative catalytic residue of TDP2 for 3′-TDP activity.***A*, catalytic mechanism underlying the 5′-TDP activity of TDP2. Nucleophilic water is activated by Asp262. Glu152 and Asp350 act as a structural nexus linking Mg^2+^ to the 5′-phosphate of DNA. Mg^2+^ mechanism is assisted by phosphotyrosyl-Arg206 cation–π and terminal deoxyribose sugar–Trp297 interfaces. *B*, the 5′-TDP activity of mutant TDP2 proteins. Data for the indicated proteins were obtained as described in Fig. 2*C*. *C*, the 3′-TDP activity of mutant TDP2 proteins. Data for the indicated proteins were obtained as described in Figure 3*A*. TDP, tyrosyl-DNA phosphodiesterase.

The catalytic mechanism underlying the 5′-TDP activity of TDP2 was elucidated in a previous study (Fig. 4*A*) (28). Asp262 activates a water molecule, which in turn induces a nucleophilic attack on the incoming 5′-phosphate of 5′-YP. Glu152 and Asp350 stabilize Mg^2+^, forming the optimal position for an interaction with 5′-phosphate. This stabilization is linked to the active site through a hydrogen bond between Trp297 and Mg^2+^ coordinating Asp350. Arg206 is critical for the formation of a TOP2 tyrosine-binding pocket with a cation–π interaction. Given the catalytic mechanism underlying 5′-TDP activity, we assessed the roles of these catalytically important amino acid residues in the 3′-TDP activity of TDP2. To this end, D262N, R206A, W297A, and D350N mutant TDP2 proteins were expressed in *E. coli* and purified to apparent homogeneity (Fig. S9). Although our analyses were not conclusive, these mutations would be unlikely to affect the protein folding of TDP2 according to homology modeling (Fig. S10). The TDP2 WT and D262N, R206A, W297A, and D350N mutant proteins were incubated with the 5′-YP (control) and 3′-YP substrates, and the products were analyzed using denaturing PAGE. The mutations completely abolished not only the 5′-TDP activity but also the 3′-TDP activity of TDP2 (Fig. 4, *B* and *C*). Thus, a similar catalytic mechanism apparently underlies the 5′-TDP and 3′-TDP activities of TDP2.

### Importance of Glu152 of TDP2 in the cellular response to CPT and ETP treatment

**Figure 5:**
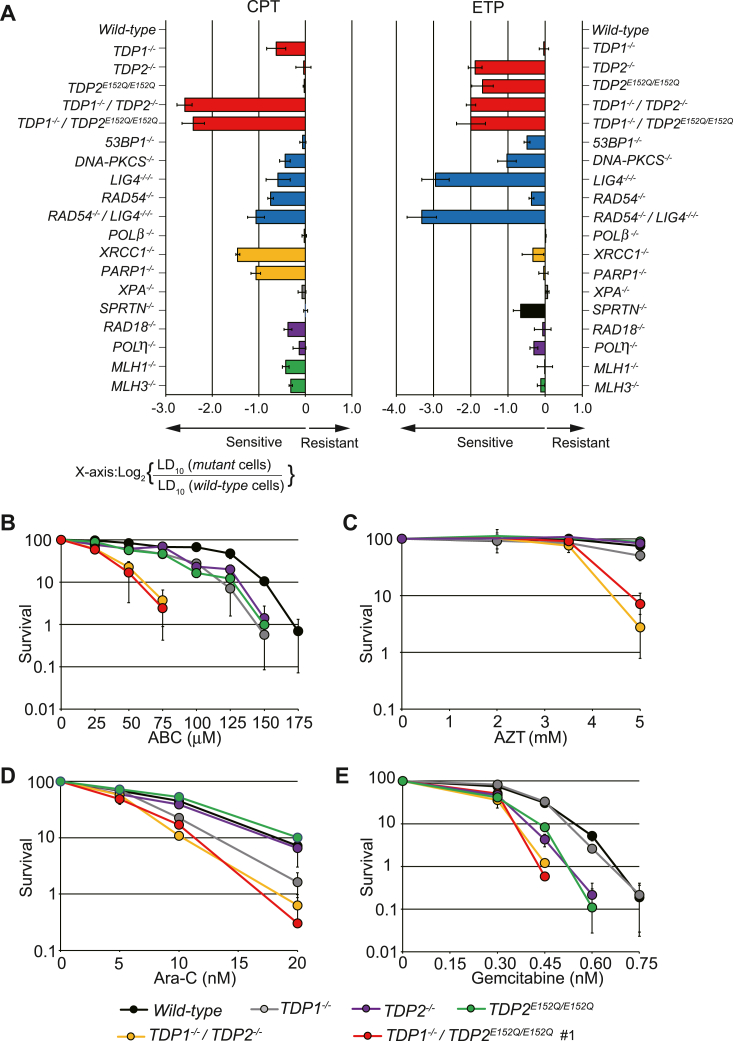
**Sensitivity of DNA repair–deficient cells to CPT, ETP, and CTNAs that produce 3′-blocking DNA lesions.***A*, CPT and ETP sensitivity profiles of selected DNA repair–deficient TK6 cells. Sensitivity of the mutant relative to the WT was determined as described in the Experimental procedures. Negative and positive scores indicate the sensitivity and resistance of a given cell line to the drug, respectively. Relative sensitivity was calculated as follows: log_2_ [(LD_10_ in *mutant* cells)/(LD_10_ in WT cells)]. Each bar is colored according to the DNA repair function category: *red*, TDP-related repair; *blue*, DSB repair; *yellow*, base excision repair (BER); *gray*, nucleotide excision repair (NER); *black*, DNA–protein crosslinks repair; *purple*, postreplication repair; and *green*, mismatch repair (MMR). Error bars are SDs of the mean of three independent assays. *B*–*E*, sensitivity of *TDP1*^*−/−*^*TDP2*^*E152Q/E152Q*^ cells to CTNAs that produce 3′-blocking DNA lesions. Indicated cells were treated with ABC (*B*), AZT (*C*), Ara-C (*D*), or gemcitabine (*E*). Survival rates were measured using colony-forming assays (as shown in Fig. 2*G*). ABC, abacavir; AZT, 3′-azido-3′-deoxythymidine; CPT, camptothecin; CTNA, chain-terminating nucleoside analog; DSB, double-strand break; ETP, etoposide; TDP, tyrosyl-DNA phosphodiesterase.

Given the widespread use of TOP1 and TOP2 inhibitors in cancer therapy, the data obtained in the present study raise the possibility that TDP2 is a critical etiological factor in the response of tumors to TOP1 and TOP2 inhibitors. Various DNA repair pathways operate during TOP1cc or TOP2cc repair (36). Therefore, understanding the relative influence of a mutation (such as E152Q) that ablates the binding of catalytic Mg^2+^ to TDP2 would provide new insights into the *in vivo* repair pathways of CPT- and ETP-induced DNA damage. TK6 cells can provide a collection of isogenic DNA repair mutant clones (37, 38); thus, we examined the CPT and ETP sensitivity profiles of a panel of DNA repair–deficient TK6 cell lines (Table 1; 19 mutant cell lines, including *TDP2*^*E152Q/E152Q*^ and *TDP1*^*−/−*^*/TDP2*^*E152Q/E152Q*^ cells), and we calculated the lethal doses for the 10% survival (LD_10_) of these cell lines. Figure 5*A* shows the ratio of the LD_10_ of individual isogenic mutants relative to the LD_10_ of WT cells on a logarithmic scale. Notably, the loss or the E152Q mutation of TDP2 led to marked increases in CPT sensitivity in the absence of TDP1 (*i.e.*, *TDP1*^*−/−*^*/TDP2*^*−/−*^ and *TDP1*^*−/−*^*/TDP2*^*E152Q/E152Q*^; Fig. 5*A*, red bars). Therefore, TDP-related repair contributes most to the repair of TOP1cc among the tested panel of cells. TOP1-induced SSBs are converted to DSBs following their collision with a replication fork (39). As expected, the DSB repair–deficient cell lines *DNA-PKCS*^*−/−*^, *LIG4*^*−/−/−*^, *RAD54*^*−/−*^, and *RAD54*^*−/−*^*/LIG4*^−/−/−^ (Fig. 5*A*, blue bars) were sensitive to CPT. Poly(ADP-ribose) polymerase 1 (PARP1) poly(ADP-ribosyl)ates TDP1 and promotes its recruitment to TOP1-induced DNA damage sites (40). Subsequently, PARylated TDP1 promotes the recruitment of XRCC1 to the TOP1-induced DNA damage sites (40), and XRCC1 acts as scaffold protein in the gap-filling repair process. Consistent with these roles, *PARP1*^*−/−*^ and *XRCC1*^*−/−*^ cells were hypersensitive to CPT (Fig. 5*A*, yellow bars).

**Table 1:** Panel of cell lines used in this study

Genotype	Function of deleted gene(s)	Reference
*TDP1*^*−/−*^	TDP-related repair	(51)
*TDP2*^*−/−*^	TDP-related repair	(24, 51)
*TDP2*^*E152Q/E152Q*^	TDP-related repair	Present study
*TDP1*^*−/−*^*/TDP2*^*−/−*^	TDP-related repair	(30, 41)
*TDP1*^*−/−*^*/TDP2*^*E152Q/E152Q*^	TDP-related repair	Present study
*53BP1*^*−/−*^	DSB repair (NHEJ)	(71)
*DNA-PKCS*^*−/−*^	DSB repair (NHEJ)	(72)
*LIG4*^*−/−/−*^	DSB repair (NHEJ)	(72)
*RAD54*^*−/−*^	DSB repair (HR)	(72)
*RAD54*^*−/−*^*/LIG4*^*−/−/−*^	DSB repair (HR/NHEJ)	(72)
*POLβ*^*−/−*^	BER	(41)
*XRCC1*^*−/−*^	BER	(37)
*PARP1*^*−/−*^	BER	(41)
*XPA*^*−/−*^	NER	(73)
*SPRTN*^*−/−*^	DPC repair, TLS	(74)
*RAD18*^*−/−*^	TLS	(47)
*POLη*^*−/−*^	TLS	(69)
*MLH1*^*−/−*^	MMR	(75)
*MLH3*^*−/−*^	MMR	(75)

BER, base excision repair; NER, nucleotide excision repair; DPC, DNA–protein crosslinks; MMR, mismatch repair; NHEJ, nonhomologous end-joining.

The 5′-phosphotyrosyl end of the TOP2-induced DSB can be converted into the 5′-phosphate end, which serves as a direct substrate for ligation by core nonhomologous end-joining machinery (41, 42, 43). Consistent with these previous findings, the nonhomologous end-joining–deficient cell lines *DNA-PKCS*^*−/−*^ and *LIG4*^−/−/−^ exhibited marked increases in ETP sensitivity. SPRTN is a DNA-dependent metalloprotease that degrades proteins in DNA–protein crosslinks, which are produced by formaldehyde and ETP (38, 44, 45, 46). *SPRTN*^*−/−*^ cells exhibited mild sensitivity to ETP but not CPT, indicating that SPRTN might contribute to the repair of TOP2ccs but not TOP1ccs. Collectively, these results suggest that TDP1/2-related repair plays critical roles in the repair of TOP1ccs and TOP2ccs, especially in comparison to SPRTN-related repair.

### Critical role played by Glu152 of TDP2 in resistance to CTNAs

We and other groups have reported that TDP1 can eliminate CTNAs from the 3′-ends of primer DNA; thus, TDP1 plays a role in cellular tolerance to CTNAs, including abacavir (ABC), 3′-azido-3′-deoxythymidine (AZT), cytarabine (Ara-C) (17, 47). The present study shows that the Mg^2+^ binding of TDP2 is essential for compensating for the loss of TDP1 in cells, that is, it ensures that the repair of the 3′-phosphotyrosyl terminus constituting a 3′-blocking lesion can continue (Fig. 3). We further investigated the role of Mg^2+^–TDP2 binding in the repair of 3′-blocking lesions by measuring the sensitivity of WT, *TDP1*^*−/−*^, *TDP2*^*−/−*^, *TDP2*^*E152Q/E152Q*^, *TDP1*^*−/−*^*/TDP2*^*−/−*^, and *TDP1*^*−/−*^*/TDP2*^*E152Q/E152Q*^ TK6 cells to CTNAs. Compared with the single mutants (*i.e.*, *TDP1*^*−/−*^ and *TDP2*^*−/−*^ cells), *TDP1*^*−/−*^*/TDP2*^*−/−*^ cells were more sensitive to ABC, AZT, Ara-C, and gemcitabine (Fig. 5, *B*–*E*), which was consistent with our previous findings (30). Likewise, *TDP1*^*−/−*^*/TDP2*^*E152Q/E152Q*^ cells were more sensitive than the single mutants (*TDP1*^*−/−*^ and *TDP2*^*E152Q/E152Q*^ cells) to ABC, AZT, Ara-C, and gemcitabine. The CTNA sensitivity of *TDP1*^*−/−*^*/TDP2*^*E152Q/E152Q*^ cells was comparable to that of *TDP1*^*−/−*^*/TDP2*^*−/−*^ cells. Therefore, the Mg^2+^ binding of TDP2 is apparently critical for the repair of various types of 3′-blocking lesions as well as TOP1ccs and TOP2ccs.

## Discussion

TDP2 possesses 5′-TDP and 3′-TDP activities for the TOP2cc and TOP1cc, respectively. The present study provides compelling genetic evidence supporting the notion that Glu152 of TDP2 is crucial for the efficient repair of the TOP2cc. We found that *TDP2*^*E152Q/E152Q*^ and *TDP2*^*−/−*^ cells exhibited the same phenotype in terms of hypersensitivity to ETP (Fig. 2*G*) and the defective repair of TOP2cc-induced DSBs in ETP-treated cells (Fig. 2*H*). Therefore, we conclude that Glu152 of TDP2 plays a critical role in the repair of the TOP2cc *in vivo*. Mechanistic analysis of the TDP2 reaction indicated that Glu152 interacts with the 5′-phosphate *via* a coordinated Mg^2+^, thereby forming the pretransition state intermediate (28, 29). According to X-ray crystal structure analysis, the clear density of the tyrosine moiety was not observed when soaking was conducted in the presence of EDTA or with crystals grown using the E161A mutant of zebrafish TDP2 protein (corresponding to E152A in humans) (27). Thus, the mutation of E152Q causes the displacement of the 5′-phosphotyrosyl-DNA substrate, resulting in defective hydrolysis of the 5′-phosphotyrosyl bond. Taken together, these findings suggest that an intact active site with a bound Mg^2+^ is required for hydrolysis of the 5′-phosphotyrosyl bond of the TOP2cc DNA substrate *in vitro* and *in vivo*.

**Figure 6:**
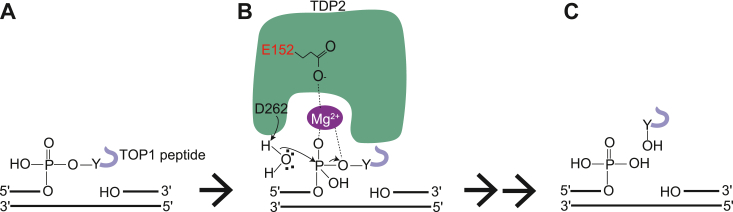
**Proposed mechanism by which the 3′-TDP activity of TDP2 hydrolyzes the 3′-phosphotyrosyl bond.***A*, in the first step of the TOP1cc repair process, the proteasomal degradation of TOP1cc yields the TOP1-derived peptide covalently attached to the 3′-end of DNA through the phosphotyrosine bond. *B*, similar to 5′-TDP activity (Fig. S1), Glu152 of TDP2 interacts with the 3′-phosphate *via* a coordinated Mg^2+^, thereby forming a pretransition state. Mg^2+^ stabilizes the tyrosine oxy-anion and the carboxylate group of Glu152 in TDP2. A water molecule is activated (possibly by Asp262 or other amino acids) and attacks the phosphate group to break the P-O bond of the tyrosine adduct. *C*, the rupture of the P-O bond of the tyrosine adduct leads to the release of the TOP1-derived peptide and the formation of the exposed 3′-phosphate end. TDP, tyrosyl-DNA phosphodiesterase; TOP, topoisomerase; TOP1cc, TOP1 cleavage complex.

The TOP1cc is repaired *via* a two-step pathway involving (i) the proteasomal degradation of the TOP1cc to the crosslinked peptide and (ii) the removal of the resulting peptide from DNA *via* the 3′-TDP activity of TDP1 (Fig. 3*C*). We previously developed an assay system for investigating the repair kinetics of the TOP1cc in the first and second steps separately (30). Using this system, we revealed that TDP2 promotes the second but not the first step of the TOP1cc repair process when TDP1 is absent, indicating that TDP2 (*i.e.*, 3′-TDP activity) functions as a backup enzyme for TDP1. In the present study, we demonstrated that the E152Q mutation of *TDP2* impairs the second step in the TOP1cc repair pathway in the absence of TDP1 (Fig. 3*G*). Consequently, *TDP1*^*−/−*^*/TDP2*^*E152Q/E152Q*^ and *TDP1*^*−/−*^*/TDP2*^*−/−*^ cells exhibited the same phenotype in terms of hypersensitivity to CPT (Fig. 3*B*) and the defective repair of TOP1cc-induced SSBs (Figs. 3*E* and S7). Our findings demonstrate that Glu152 of TDP2 is critical for the hydrolysis of the 3′-phosphotyrosyl bond after the proteasomal degradation of the TOP1cc. The 3′- and 5′-TDP activities of TDP2 have common divalent metal ion requirements (Fig. 1, *D* and *E*). Additionally, mutations at residues Asp262, Trp297, Arg206, or Asp350 abolished both the 3′-TDP and 5′-TDP activities of TDP2 (Fig. 4, *B* and *C*), suggesting that a common catalytic mechanism underlines the 3′-TDP and 5′-TDP activities of TDP2. Based on these findings, we propose a model of the catalytic mechanism underlying TOP1cc repair (*i.e.*, 3′-TDP activity) by TDP2. In the first step of TOP1cc repair, the proteasomal degradation of the TOP1cc yields the TOP1-derived peptide attached to the 3′-end of DNA through the phosphotyrosine bond (Fig. 6*A*). We assume that, similar to 5′-TDP activity (Fig. S1), Glu152 of TDP2 interacts with the 3′-phosphate *via* a coordinated Mg^2+^, thereby forming a pretransition state (Fig. 6*B*). An activated water molecule attacks the phosphate group, leading to the release of the TOP1-derived peptide and the formation of the exposed 3′-phosphate end (Fig. 6*C*). It remains unclear how TDP2 accommodates the DNA ends with opposite polarities (*i.e.*, the 3′ DNA end in Fig. 6*B* and 5′ DNA end in Fig. S1*B*) at the same active site, exerting both 3′-TDP and 5′-TDP activities.

Compared with WT cells, *TDP2*^*E152Q/E152Q*^ cells unexpectedly exhibited a small but significant delay in the kinetics of the second step of the TOP1cc repair process (15 and 30 min repair; Fig. 3*G*). Such delayed kinetics were not observed when *TDP2*^*−/−*^ cells were tested (Fig. 3*G*). This may be due to the inactive E152Q mutant of TDP2 competing with TDP1 for the damage site of the CPT-induced TOP1cc, thereby inhibiting the repair action of TDP1 in the presence of TDP1. However, the apparent repair rate of *TDP2*^*E152Q/E152Q*^ cells was comparable to that of WT cells at 60 min, indicating that the E152Q mutation barely affects TOP1cc repair in the second step of the pathway. Therefore, the E152Q mutation of TDP2 did not cause a detectable increase in cellular sensitivity to CPT (Fig. 3*B*).

SPRTN functions as a metalloprotease that degrades DNA–protein crosslinks, which can originate from the covalent stabilization of enzyme–DNA intermediates (*e.g.*, TOP1ccs and TOP2ccs) or the chemical crosslinking of DNA-binding proteins to DNA (*e.g.*, formaldehyde-induced DNA–protein crosslinks) (48). Thus, the degradation of the TOP1cc and TOP2cc to the corresponding peptide crosslinks by SPRTN instead of proteasomes followed by the removal of the peptide crosslinks by TDP1/TDP2 could constitute an alternative repair pathway for TOP1ccs and TOP2ccs. However, relative to cells deficient in TDP1/TDP2-related repair, *SPRTN*^*−/−*^ cells exhibited marginal and mild sensitivities to CPT and ETP, respectively (Fig. 5*A*). Accordingly, TOP1cc and TOP2cc repair likely occurs more through the proteasome–TDP1/TDP2-based repair pathway than through the SPRTN–TDP1/TDP2-based repair pathway. In addition to the aforementioned repair pathways, TOP1ccs and TOP2ccs can also be removed by endonucleases that incise the DNA flanking the complexes. Some nucleases, such as the MRN complex and CtIP, may also be involved in this process (49, 50); however, we did not include these nuclease-related mutants in the panel of tested cells because they are lethal (51, 52).

CTNAs have been widely used for treating cancer and viral infections. ABC and AZT are antiviral nucleotide analogs. They are phosphorylated and misincorporated into genomic DNA by replicative DNA polymerases, resulting in the generation of 3′-blocking damages. TDP1 has broad substrate specificity (13) and 3′-nucleosidase activity, which can remove incorporated ABC and AZT (17). In contrast to the broad substrate specificity of TDP1, it was believed until recently that the repair activity of TDP2 was reserved specifically for substrates containing 5′-phosphotyrosyl bonds (25). We found previously that *TDP2*^*−/−*^ cells are sensitive to ABC and AZT (30). Thus, TDP1 and TDP2 concurrently contribute to the repair of ABC- and AZT-induced DNA damage (Fig. 5, *B* and *C*). Ara-C and gemcitabine are anticancer chemotherapeutic drugs. They are also frequently incorporated into genomic DNA by the replicative polymerase. Although Ara-C acts by blocking extension of the nascent DNA strand (47), gemcitabine stops extension after incorporation of additional nucleotides (53). Consistent with previous data, *TDP1*^*−/−*^/*TDP2*^*−/−*^ cells were more sensitive to Ara-C than *TDP1*^*−/−*^ cells, whereas *TDP2*^*−/−*^ cells showed no sensitivity to this agent (Fig. 5*D*). On the contrary, *TDP1*^*−/−*^ cells were not sensitive to gemcitabine but *TDP2*^*−/−*^ cells were (Fig. 5*E*). These data indicate that TDP1 seems more important in removing incorporated Ara-C, while TDP2 seems more important in removing incorporated gemcitabine. In the present study, the CTNA sensitivity profile of *TDP2*^*E152Q/E152Q*^ cells was essentially the same as that of *TDP2*^*−/−*^ cells (Fig. 5, *B*–*E*). Likewise, *TDP1*^*−/−*^*/TDP2*^*E152Q/E152Q*^ and *TDP1*^*−/−*^/*TDP2*^*−/−*^ cells exhibited similar phenotypes in terms of CTNA sensitivity. These findings raise the possibility that the Mg^2+^ binding of TDP2 is critical for 3′-nucleosidase activity and removing various 3′-blocking lesions generated by CTNAs. Meanwhile, Ara-C and gemcitabine treatment enhances the trapping of TOP1cc (54, 55). Thus, we cannot rule out the possibility that the CTNA sensitivity profiles in *TDP1*^*−/−*^ and *TDP2*^*−/−*^ cells reflect different levels of TOP1 trapping by CTNA. However, these mechanisms are speculative and further studies are necessary.

Focusing on the broad substrate spectrum of TDP2, the inhibition of TDP2 is an attractive target for tumor cell sensitization in combination with TOP1 inhibitors and CTNAs. Some studies have reported the development of specific TDP2 inhibitors, which include deazaflavin (56, 57, 58, 59), isoquinoline-1,3-dione (60), furoquinoline (61), diaminoquinoline-2,8-dione (62), and isoxazoloquinolinedione (63). In addition, the derivatives of indenoisoquinoline were found to be triple TOP1–TDP1–TDP2 inhibitors (64). These compounds inhibit TDP2 mainly by occupying its DNA-binding groove rather than directly inactivating its catalytic activity (65). Isoquinoline-1,3-dione and 4-benzylideneisoquinoline-1,3(2H,4H)-dione have been found to coordinate with Glu152 and Mg^2+^ (60, 66). The present study shows that the inhibition of Mg^2+^ binding completely abolishes the 3′- and 5′-TDP activity of TDP2 *in vitro* and *in vivo*. Thus, increasing Mg^2+^ chelating efficiency or completely breaking the coordination of Glu152 and Mg^2+^ could lead to the development of TDP2 inhibitors with higher efficacies and should be tested accordingly. It is also conceivable that the use of such TDP2 inhibitors will have immense clinical benefits for cancer treatment.

## Experimental procedures

### Materials

Anti-TOP1 antibody [EPR5375] (ab109374) was purchased from Abcam. Anti-TDP2 antibody (sc-377280) was obtained from Santa Cruz, and anti-GAPDH antibody (60004-1-lg) was obtained from Proteintech. Horseradish peroxidase (HRP)-conjugated antimouse IgG (ab6789) and HRP-conjugated antirabbit IgG (ab6721) antibodies were purchased from Abcam. Anti-TOP1cc (clone 1.1A; MABE1084) was purchased from Merck. CPT and ETP were obtained from Wako. TDP1 recombinant protein with an N-terminal GST tag (ab131921) was purchased from Abcam. ABC (A2694), AZT (A2052), Ara-C (C2035), and gemcitabine (G0544) were purchased from Tokyo Chemical Industry.

### Preparation of oligonucleotide substrates

The 3′-YP oligonucleotide (5′-TCCGTTGAAGCCTGCTTT-Tyr-3′) was prepared as reported previously (67). The 3′-YP oligonucleotide was 5′-labeled with ^32^P using [γ-^32^P]ATP and T4 polynucleotide kinase (2021S, TAKARA). For the blunt-end DNA substrate bearing 3′-pTyr, a radiolabeled 3′-YP (above) was annealed with a 2-fold molar excess of a 18-bp complementary oligonucleotide (5′-AAAGCAGGCTTCAACGGA-3′). For the 5′-overhang DNA substrate with 3′-pTyr on the recessed 3′ end, a radiolabeled 3′-YP (above) was annealed with a 2-fold molar excess of partly complementary 36-bp oligonucleotide (5′-TCTACAGACATCATCGGTAAAGCAGGCTTCAACGGA-3′). For the nicked duplex DNA substrate containing 3′-pTyr at the nick, a radiolabeled 3′-YP (above) was annealed with a 2-fold molar excess of a 36-bp oligonucleotide (5′-TCTACAGACATCATCGGTAAAGCAGGCTTCAACGGA-3′) and a 4-fold molar excess of a 18-bp oligonucleotide (5′-ACCGATGATGTCTGTAGA-3′). For the duplex 5′-phosphotyrosyl substrate, the 5′-YP oligonucleotide (5′-Tyr-TCCGTTGAAGCCTGCTTT-3′), which was kindly gifted by Dr Shunichi Takeda, was annealed with a complementary oligonucleotide (5′-GAAAGCAGGCTTCAACGGA-3′), and the resulting one nucleotide 5′ overhang was filled in with [α-^32^P]dCTP and a Klenow fragment (3′→5′ exo^−^) (M0212S, New England Biolabs). All other oligonucleotides were synthesized by Eurofins.

### Cloning and purification of recombinant human TDP2

Total RNA was extracted from WT or *TDP2*^*E152Q/E152Q*^ TK6 cells, and first-strand complementary DNA was prepared using a Superscript III First-Strand Synthesis System (18080051, Invitrogen). WT or *mutant* human *TDP2* DNA was amplified from the first-strand complementary DNA using the primers 5′-GGGCATATGGAGTTGGGGAGTTGCCTGGAGGGC-3′ and 5′-GGGGGATCCTTACAATATTATATCTAAGTTGCA-3′, which enabled the introduction of *Nde*I and *Bam*HI sites at 5′ and 3′ positions, respectively. The PCR products were digested with *Nde*I and *BamH*I and cloned into the expression vector pET16b at the *Nde*I/*BamH*I site, enabling the expression of the TDP2 protein with a 10× His tag. D262N, R206A, W297A, and D350N mutations were introduced into the pET16b-TDP2 (WT) vector using a KOD-Plus-Mutagenesis Kit (SMK101/DNA903F, TOYOBO). The primers used for site-directed mutagenesis are listed in Table S1. The validity of the constructs was confirmed *via*DNA sequencing. *E. coli* BL21(DE3) cells carrying the expression vector of TDP2 (WT or mutants) were cultured in LB media at 37 °C until the absorbance at 600 nm reached 0.2, after which the culture was cooled to 25 °C. After IPTG was added to 1 mM, the cells were cultured at 25 °C for 16 h, harvested *via* centrifugation, and resuspended in His60 Ni × Tractor buffer (635665, TAKARA) with the addition of protease inhibitors, lysozyme, and Benzonase. Subsequently, the cells were lysed *via* incubation on ice for 10 min, and the lysate was clarified using centrifugation at 15,000 rpm and 4 °C for 20 min. The supernatant was applied to a 1 ml His60 Ni Gravity Column (635657, TAKARA) that was pre-equilibrated with His60 Ni equilibration buffer (50 mM sodium phosphate, 300 mM NaCl, and 20 mM imidazole; pH 7.4). The column was washed with 10 ml of wash buffer (50 mM sodium phosphate, 300 mM NaCl, and 40 mM imidazole; pH 7.4) and eluted with elution buffer (50 mM sodium phosphate, 300 mM NaCl, and 300 mM imidazole; pH 7.4). The peak fractions containing the recombinant protein, which was detected using SDS-PAGE, were pooled and dialyzed against storage buffer [25 mM Tris–HCl (pH 7.5), 100 mM NaCl, 1 mM DTT, and 10% glycerol]. The protein was stored at −80 °C in aliquots until future use.

### *In vitro* 3′- and 5′-TDP activity assays

5′- or 3′-^32^P–labeled DNA substrates were incubated with the indicated concentrations of recombinant human TDP1 or TDP2 for 10 min at 37 °C in 5 μl of reaction buffer. The composition of the TDP reaction buffer was 25 mM Hepes–NaOH (pH 8.0), 1 mM DTT, and 40 μg/ml bovine serum albumin unless otherwise indicated. Reactions were terminated by adding one volume of gel loading buffer (formamide containing 2.5 mM EDTA). Samples were separated using 20% denaturing polyacrylamide gel containing 7 M urea in TBE buffer (89 mM Tris, 89 mM boric acid, and 2 mM EDTA). Following electrophoresis, the radioactivity of the gel was measured using a Typhoon FLA9500 (GE Healthcare Life Sciences).

### Structural modeling

Structural models were generated using the SWISS-MODEL server with reference to the crystal structure of the human TDP2 mutants of the WT (PDB: 5INO). The superpositions of these structures were determined using UCSF ChimeraX.

### TK6 cell culture

Human lymphoblast TK6 cells (68) were provided by Dr Shunichi Takeda and Dr Hiroyuki Sasanuma (Department of Radiation Genetics, Graduate School of Medicine, Kyoto University; Table 1). The cells were cultured as described previously (38, 69), that is, they were cultured in RPMI 1640 medium (189-02025, Wako) supplemented with 5% heat-inactivated horse serum, L-glutamine (16948-04, Nacalai Tesque), 0.2 mg/ml sodium pyruvate (Sigma-Aldrich), 100 U/ml penicillin, and 100 μg/ml streptomycin (168-23191, Nacalai Tesque) and maintained at 37 °C in a humidified atmosphere containing 5% CO_2_.

### Generation of *TDP2*^*E152Q/E152Q*^ and *TDP1*^*−/−*^*/TDP2*^*E152Q/E152Q*^ TK6 cells

To generate human *TDP2*^*E152Q/E152Q*^ and *TDP1*^*−/−*^*/TDP2*^*E152Q/E152Q*^ TK6 cells, we prepared the targeting construct from a genomic sequence covering the *TDP2* gene. TDP2-E152Q mutation knock-in constructs (*TDP2-HYG*^*R*^ and *TDP2*-*PURO*^*R*^) were generated from genomic PCR products combined with a resistance gene cassette (*HYG*^*R*^ and *PURO*^*R*^) flanked by loxP signals at both ends (see Results for details). The primers used to amplify the left arm were 5′-GCGAATTGGGTACCGGGCCACACACCCTCAGGAAGTCCT-3′ and 5′-CTGGGCTCGAGGGGGGGCCTTAATTCTTCAGATGTTTCT-3′, whereas those used to amplify the right arm were 5′-TGGGAAGCTTGTCGACTTAAGGTAGCTGAAAATGAAATGG-3′ and 5′-CACTAGTAGGCGCGCCTTAACATGGGAATCAGAAATCCTA-3′. The amplified fragments of the left and right arms were assembled *via* a seamless reaction (GeneArt Seamless Cloning and Assembly Kit; Invitrogen) into *DT-ApA/HYG*^*R*^ and *DT-ApA/PURO*^*R*^ vectors (both kindly gifted by Dr Shunichi Takeda and Dr Hiroyuki Sasanuma), which were predigested with *Apa*I and *Afl*II. Single and double underlining in the abovementioned primers indicates the homology upstream and downstream from the *Apa*I and *Afl*II sites, respectively. The point mutation in the right arm sequence causing the E152Q amino acid replacement was introduced *via* PCR using the following primers: 5′-TAATATGGGGGAATAACTTGCTGTAGAAATATCACATCTG-3′ and 5′-CAAGTTATTCCCCCATATTATAGCTACCTAAAGAAGAGAT-3′. In the CRISPR/Cas9 system, we used the pX330 vector (Addgene), which is designed to recognize the sequence 5′-CTCAATATTAGGTGCCCTAGAGG-3′ for *TDP2-CRISPR*. WT and *TDP1*^*−/−*^ TK6 cells were transfected with 2 μg of each targeting vector (*TDP2-HYG*^*R*^ and *TDP2*-*PURO*^*R*^) and 6 μg of the guide sequence containing the pX330 vector using the NEON Transfection System (MPK10025, Thermo Fisher Scientific) at 1350 V, 10 ms, and 3 pulses according to the manufacturer’s instructions. After 48 h, the cells were plated in 96-well plates and subjected to hygromycin (0.3 mg/ml) and puromycin (0.5 μg/ml) treatments. The drug-resistant colonies were picked 8 days after transfection. The selection marker gene was removed *via* the transient expression of CRE recombinase. Knock-in of the mutation in the marker gene pop-out cells was confirmed by sequencing the RT-PCR products.

### Sensitivity of cells to ETP and CPT

In 2 ml of media, 2 × 10^3^ cells were treated with the indicated concentrations of ETP or CPT for 3 h at 37 °C. Cells were then serially diluted and plated in triplicate into 6-well plates containing 5 ml/well of 1.5% (w/v) methylcellulose (M0387, Sigma-Aldrich) and Dulbecco’s modified Eagle’s medium F-12 (042-30555, Life Technologies) supplemented with 10% horse serum. The number of colonies was counted at day 10 to 14.

### Western blot

Cells were collected and lysed with a Laemmli sample buffer (62.5 mM Tris–HCl, 10% glycerol, and 145 mM 2-mercaptoethanol). Lysates were separated using 10% SDS-PAGE, and the separated samples were blotted on a nitrocellulose membrane. The membrane was probed with anti-TDP2 or anti-GAPDH antibody, and the primary antibodies were detected using HRP-conjugated antimouse IgG antibodies.

### Neutral comet assays

Cells were treated with 1 μM ETP for 60 min at 37 °C and then incubated in drug-free media for 30 min. Subsequently, 5.0 × 10^4^ cells were washed in prechilled PBS and mixed with 45 μl aliquots of CometAssay LMAgarose (4250-050-02, R&D Systems) at 37 °C. Cell suspensions were immediately transferred onto a CometSlide slide (4250-200-03, R&D Systems) and maintained at 4 °C for 10 min. The slides were immersed in prechilled CometAssay Lysis Solution (4250-050-01, R&D Systems) overnight, after which they were incubated for 15 min in prechilled neutral electrophoresis buffer containing 0.3 M sodium acetate and 0.1 M Tris–acetate (pH 9.0). Electrophoresis was then conducted at 30 V for 60 min using an ATTO Submarine Electrophoresis System (#AE-6125; ATTO). Subsequently, the slides were washed with DNA precipitation solution containing 1.0 M ammonium acetate and 82% ethanol for 30 min and fixed in 70% ethanol for 30 min. DNA was stained with SYBR gold (Invitrogen) diluted 1:10,000 in TE buffer (10 mM Tris–HCl and 1 mM EDTA; pH 7.5) for 30 min, and the slides were washed with water for 10 min prior to observations under a fluorescence microscope (TE2000; Nikon) at 200 × magnification. Tail moments from 50 cells/sample were measured using OpenComet software (https://cometbio.org/index.html) (70).

### Alkaline comet assays

Cells were incubated with 25 μM CPT for 60 min at 37 °C and then incubated in CPT-free media for 30 to 60 min. They were then embedded in agarose and treated with lysis buffer as described for the neutral comet assays. The produced slides were incubated for 40 min in prechilled alkaline electrophoresis buffer (0.2 M NaOH and 1 mM EDTA; pH 13). Electrophoresis was performed as described for the neutral comet assays. The slides were washed with water for 2 × 5 min and fixed in 70% ethanol for 5 min at room temperature. Finally, the slides were observed and tail moments were analyzed as described for the neutral comet assays.

### Detection of TOP1cc using slot blotting

Genomic DNA was isolated from the untreated control and CPT-treated TK6 cells using cesium chloride density gradient ultracentrifugation as described previously (24, 30). DNA was slot-blotted on a membrane, and TOP1cc was analyzed using anti-TOP1 and anti-TOP1cc primary antibodies with the HRP-conjugated secondary antibody as described previously (30).

## Data availability

Atomic coordinates for the models of mutant TDP2 generated *via* SWISS-MODEL are available as supplementary information. The original contributions presented in the study are included in the article/Supplementary information. Further inquiries related to data availability can be directed to the corresponding author.

## Supporting information

This article contains supporting information.

## Conflict of interest

The authors declare that they have no conflict of interest with the contents of this article.

## Supporting information

Supplementary Information Figure

PDB data
