# Supplementary material for: Repair of topoisomerase 1–induced DNA damage by tyrosyl-DNA phosphodiesterase 2 (TDP2) is dependent on its magnesium binding
Source: J Biol Chem. 2023 Jun 29;299(8):104988. doi: 10.1016/j.jbc.2023.104988 (PMC10407441; doi:10.1016/j.jbc.2023.104988)
Supplement: Supplementary Information Table [file mmc1.docx]

**Supplementary Table S1.** Sequences of primers.

| Primer Name | Sequence |
| --- | --- |
| TDP2(D262N) Forward | 5′-CCTTAGATTTGTATTTCCTGCAAATATAAC-3′ |
| TDP2(D262N) Reverse | 5′-GTTATATTTGCAGGAAATACAAATCTAAGG-3′ |
| TDP2(R206A) Forward | 5′-ACATAAAAGGTTTGCCATCATTTTGGTACT-3′ |
| TDP2(R206A) Reverse | 5′-AGTACCAAAATGATGGCAAACCTTTTATGT-3′ |
| TDP2(W297A) Forward | 5′-CATTTGTGTATCCGCTGTATACTGGCAATG-3′ |
| TDP2(W297A) Reverse | 5′-CATTGCCAGTATACAGCGGATACACAAATG-3′ |
| TDP2(D350N) Forward | 5′-AAGACCCCAGTGATTACTAGGAAATCTACC-3′ |
| TDP2(D350N) Reverse | 5′-GGTAGATTTCCTAGTAATCACTGGGGTCTT-3′ |
