# Supplementary material for: Repair of topoisomerase 1–induced DNA damage by tyrosyl-DNA phosphodiesterase 2 (TDP2) is dependent on its magnesium binding
Source: J Biol Chem. 2023 Jun 29;299(8):104988. doi: 10.1016/j.jbc.2023.104988 (PMC10407441; doi:10.1016/j.jbc.2023.104988)
Supplement: Supplementary Information Figure [file mmc2.pdf]

Supporting Information

Repair of topoisomerase 1-induced DNA damage by tyrosyl-DNA phosphodiesterase 2 (TDP2) is dependent on its magnesium binding

Naoto Shimizu[1], Yusaku Hamada[2], Ryosuke Morozumi[2], Junpei Yamamoto[3], Shigenori Iwai[3], Kei-ichi Sugiyama[4], Hiroshi Ide[1], Masataka Tsuda[1,2,4]

[1]Program of Mathematical and Life Sciences, Graduate School of Integrated Sciences for Life, Hiroshima University, Higashi-Hiroshima, Japan

[2]Program of Biomedical Science, Graduate School of Integrated Sciences for Life, Hiroshima University, Higashi-Hiroshima, Japan

[3]Graduate School of Engineering Science, Osaka University, 1-3 Machikaneyama, Toyonaka, Osaka, Japan

[4]Division of Genetics and Mutagenesis, National Institute of Health Sciences, Tonomachi, Kawasaki-ku, Kawasaki-shi, Kanagawa, Japan

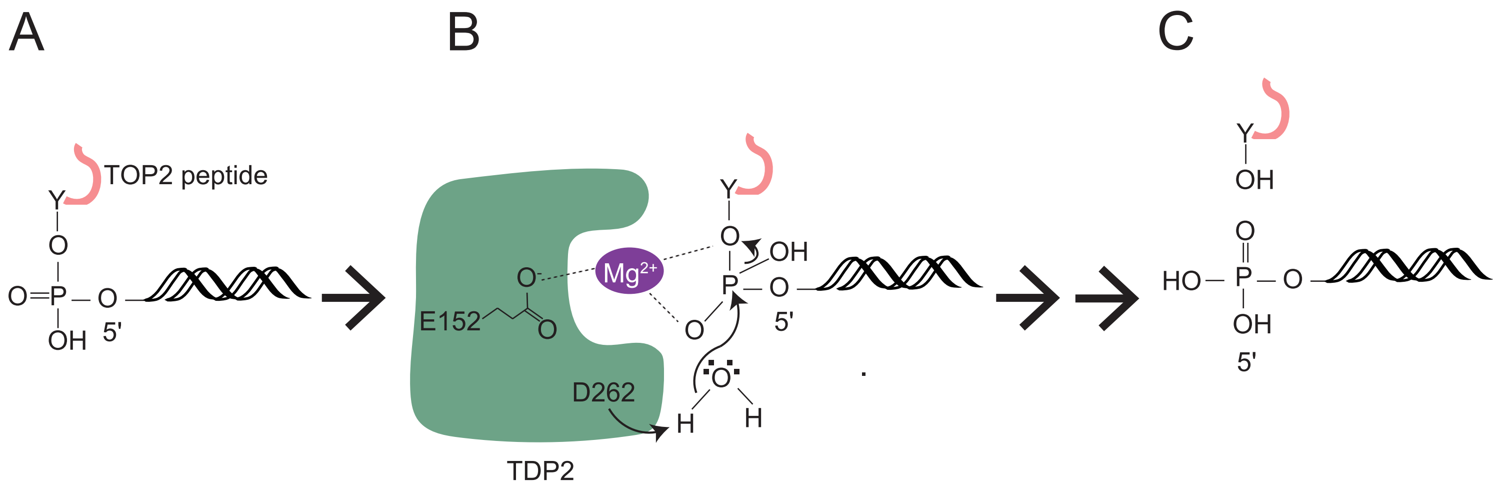

Supplementary Figure S1. Catalytic mechanism underlying the 5′-TDP activity of TDP2.

(A) In the first step of the TOP2cc repair process, the proteasomal degradation of the TOP2cc gives rise to the TOP2-derived peptide covalently attached to the 5′-end of DNA via a phosphotyrosine bond.

(B) Glu152 of TDP2 interacts with the 5′-phosphate via the coordinated Mg^{2+}, forming a pretransition state. Mg^{2+} stabilizes the oxy-anion of tyrosine and the carboxylate group of Glu152 in TDP2. A water molecule is activated by Asp262 of TDP2 and undertakes nucleophilic attacks on the phosphate group to break the P-O bond of the tyrosine adduct.

(C) The rupture of the P-O bond of the tyrosine adduct leads to the release of the TOP2-derived peptide and the formation of the exposed 5′-phosphate end.

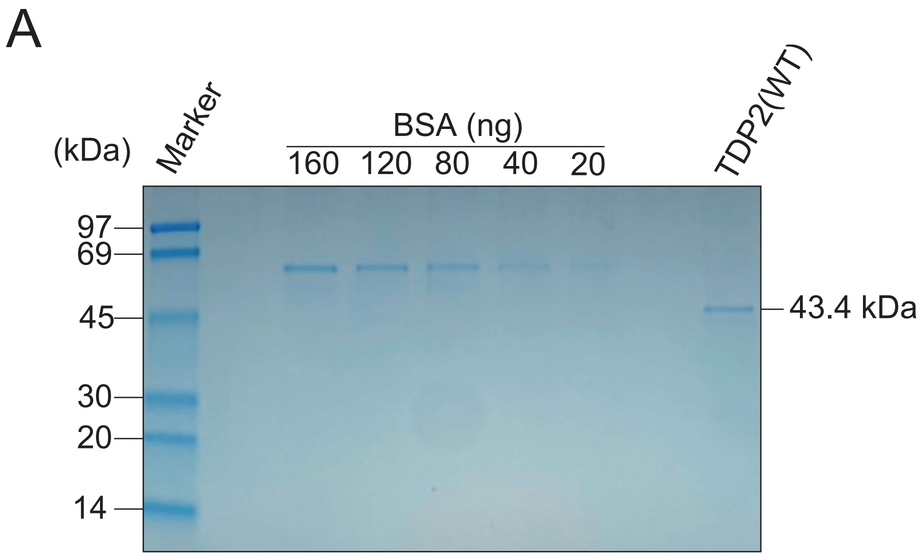

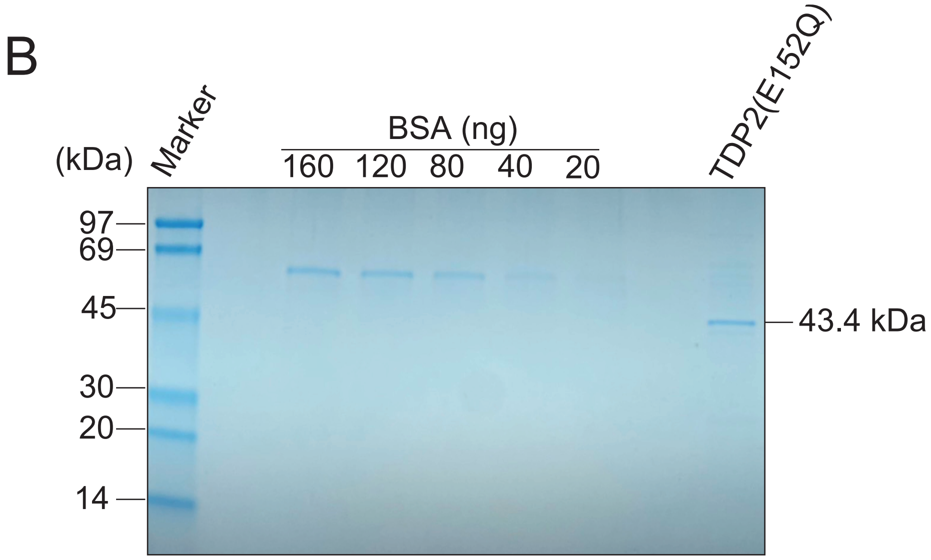

Supplementary Figure S2. SDS-PAGE analysis of the purified recombinant proteins of human TDP2 (wild-type) (A) and TDP2 (E152Q mutant) (B).

The purification process is described in the Materials and methods. Concentrations and purities of the purified proteins were estimated using the intensities of protein bands (stained with Coomassie brilliant blue) in an SDS-PAGE using bovine serum albumin as a standard. The sizes (43.4 kDa) of the recombinant proteins (wild-type and E152Q mutant) correspond to those of proteins containing 10× His-tags and the linker peptide.

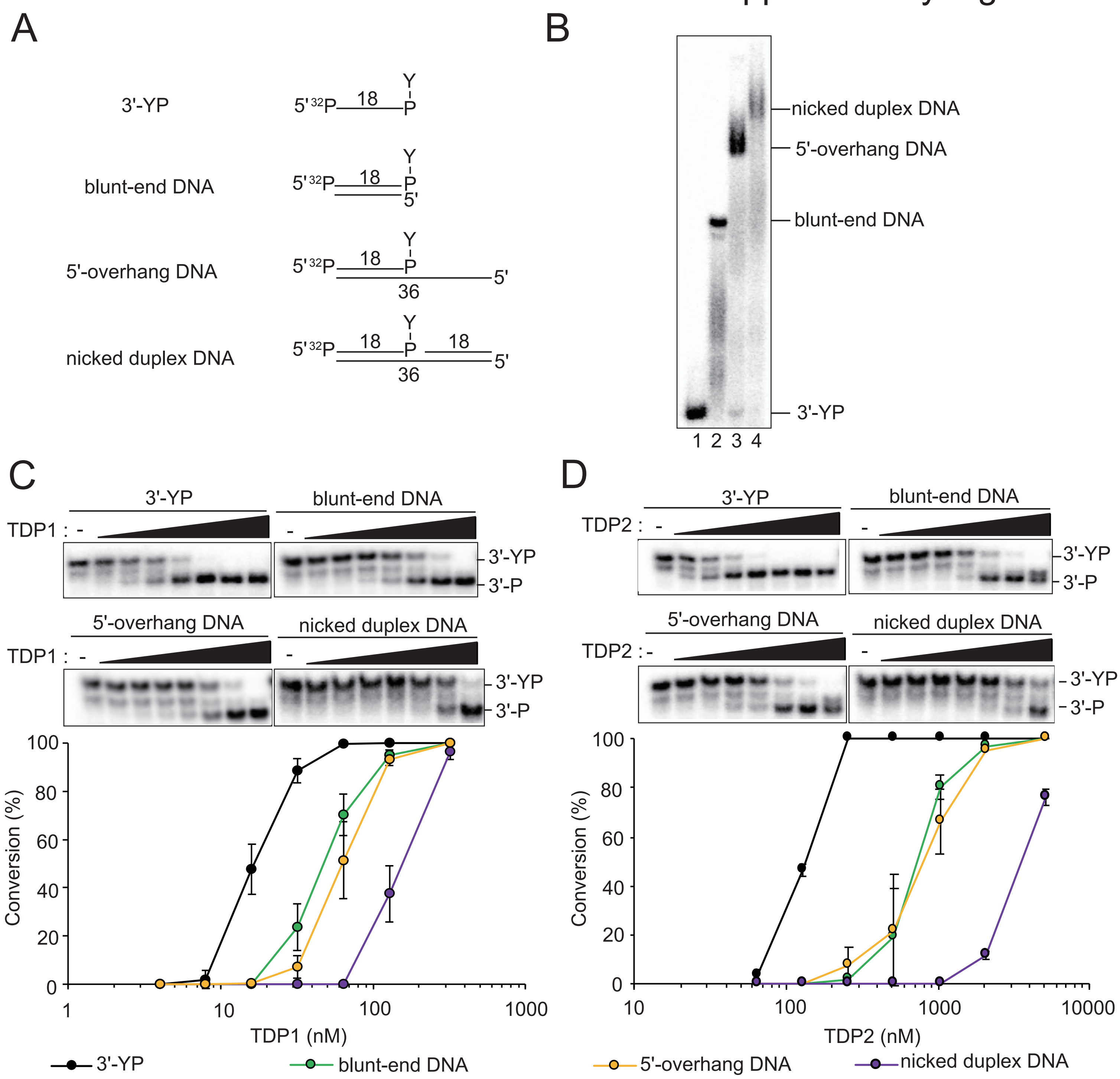

Supplementary Figure S3. Substrate specificity of TDP1 and TDP2.

(A) Schematic representation of the four types of DNA substrates bearing 3′-pTyr used in this study: single-stranded DNA (3′-YP), blunt-end DNA, 5′-overhang DNA, and nicked duplex DNA. The tyrosine in 3′-pTyr is indicated by Y. In all experiments, only the DNA strand containing the 3′-pTyr was 5′-^{32}P-radiolabeled. This oligonucleotide was annealed to complementary oligonucleotides as described under "Experimental Procedures" to generate blunt-end DNA, 5′-overhang DNA, and nicked duplex DNA substrates.

(B) 5′-^{32}P-radiolabeled 3′-YP, blunt-end DNA, 5′-overhang DNA, and nicked duplex DNA were separated by native PAGE. Lane1: 3′-YP, Lane2: blunt-end DNA Lane3: 5′-overhang, Lane4: nicked duplex DNA. The position of each substrate is indicated.

(C and D) Upper: Wild-type TDP1 (0, 4, 8, 16, 32, 64, 128, and 320 nM) (C) or TDP2 (0, 64.3, 128.5, 257, 514, 1028, 2056, and 5140 nM) (D) were incubated with the indicated substrate in a TDP assay buffer containing 1 mM Mg^{2+} at 37°C for 10 min, and the products were analyzed using denaturing PAGE. The substrate bearing 3′-pTyr and the product without Tyr are indicated by 3′-YP and 3′-P, respectively. Lower: Conversion (%) of the substrate bearing 3′-pTyr to the product without Tyr at different TDP1 and TDP2 concentrations.

Shimizu et al.
Supplementary Figure S4

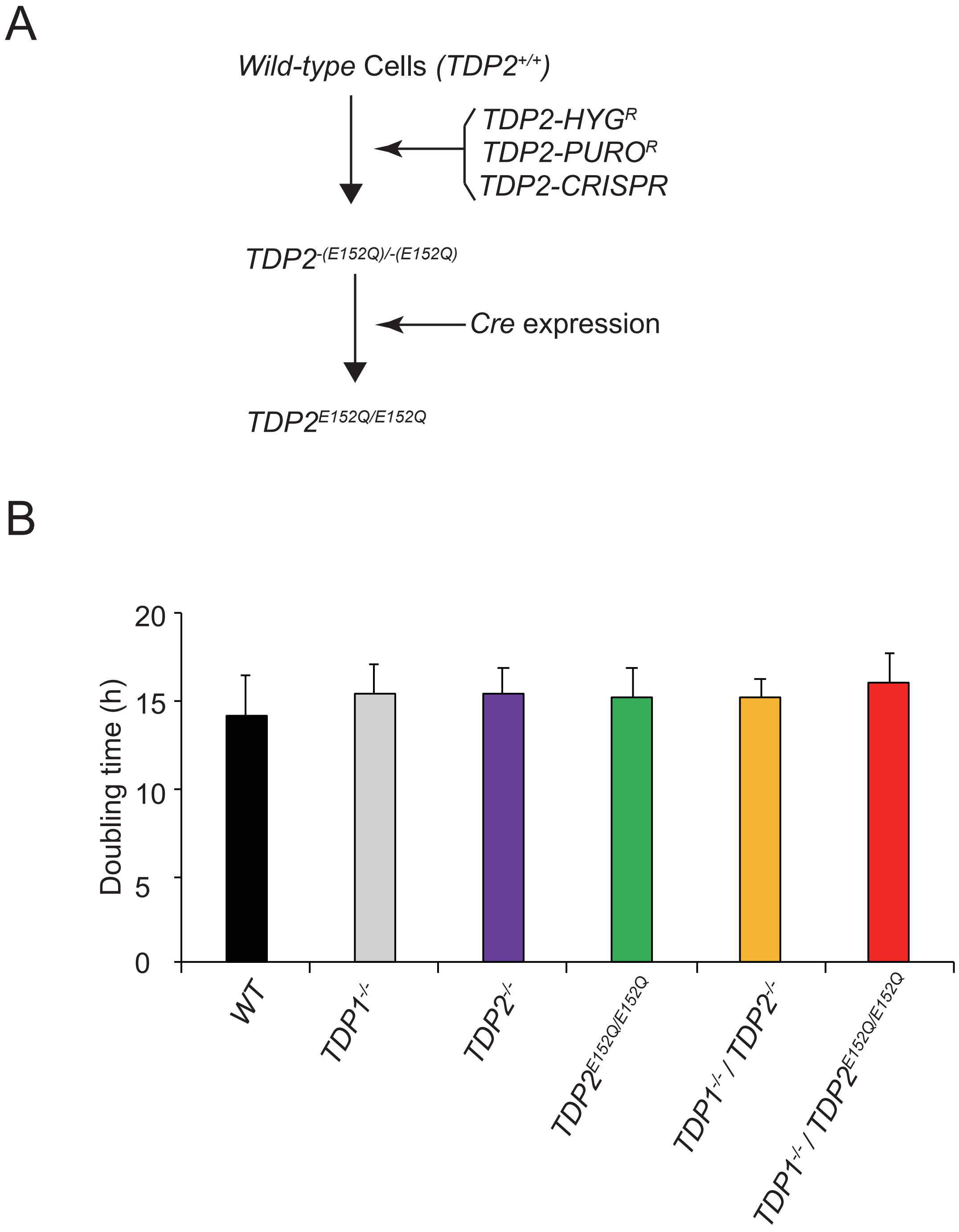

Supplementary Figure S4. Generation and growth rates of TK6 mutant cells.

(A) Outline of the strategy for targeting the TDP2 locus to generate $TDP2^{E152Q/E152Q}$ cells.

(B) Average doubling time of the indicated TK6 mutant cells. Error bars are the SDs of at least three independent experiments.

.

A

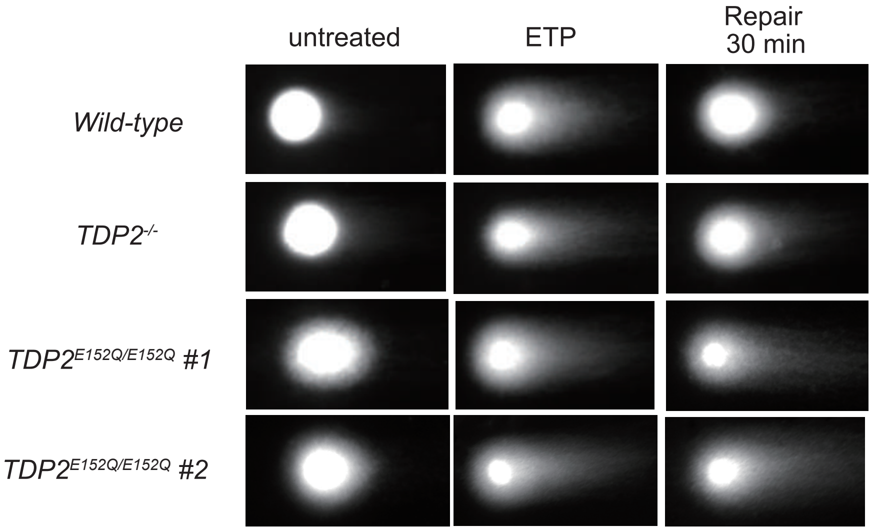
untreated
ETP
Repair
30 min
Wild-type
TDP2-/-
TDP2E152Q/E152Q #1
TDP2E152Q/E152Q #2

B

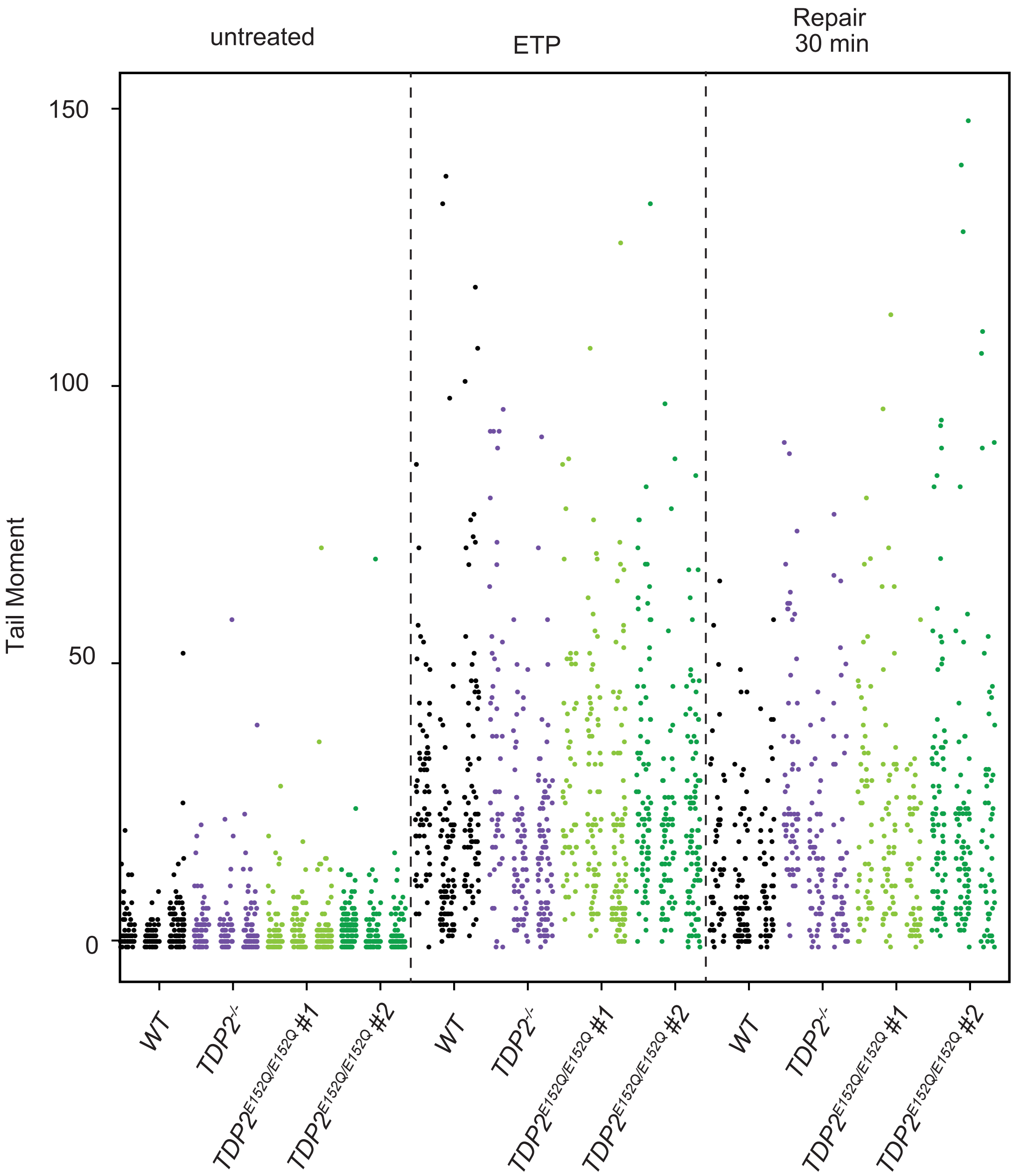
untreated
ETP
Repair
30 min
Tail Moment
150
100
50
0
WT
TDP2-/-
TDP2E152Q/E152Q #1
TDP2E152Q/E152Q #2

Supplementary Figure S5. Neutral comet assay used to measure the repair kinetics of DSBs in *TDP2*$^{-/-}$ and *TDP2*$^{E152Q/E152Q}$ cells. Indicated cells were treated with ETP or left untreated and analyzed for DSBs using neutral comet assays (as described in **Figure 2H**). Typical neutral comet images (A) and the raw data of tail moments before standardization (B) are shown. Data below ETP (A and B) indicate the samples prepared immediately after ETP treatment (0 min repair).

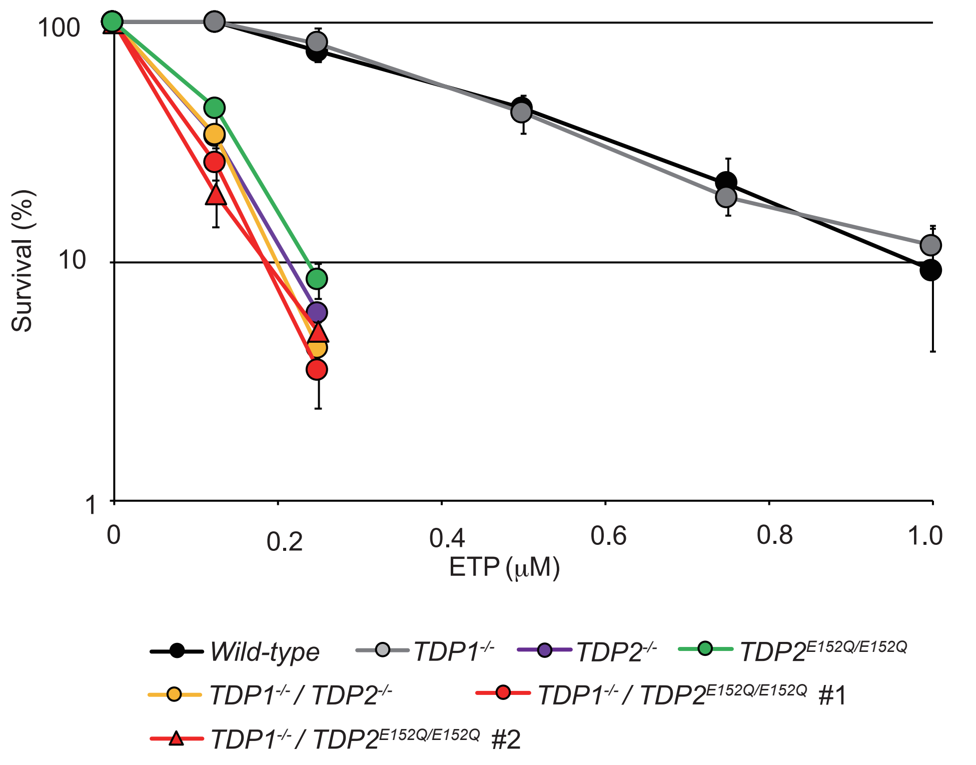

Supplementary Figure S6. Survival curves of TK6 mutant cells treated with ETP. Survival rates of the indicated cells were measured using colony forming assays (as described in Figure 2G).

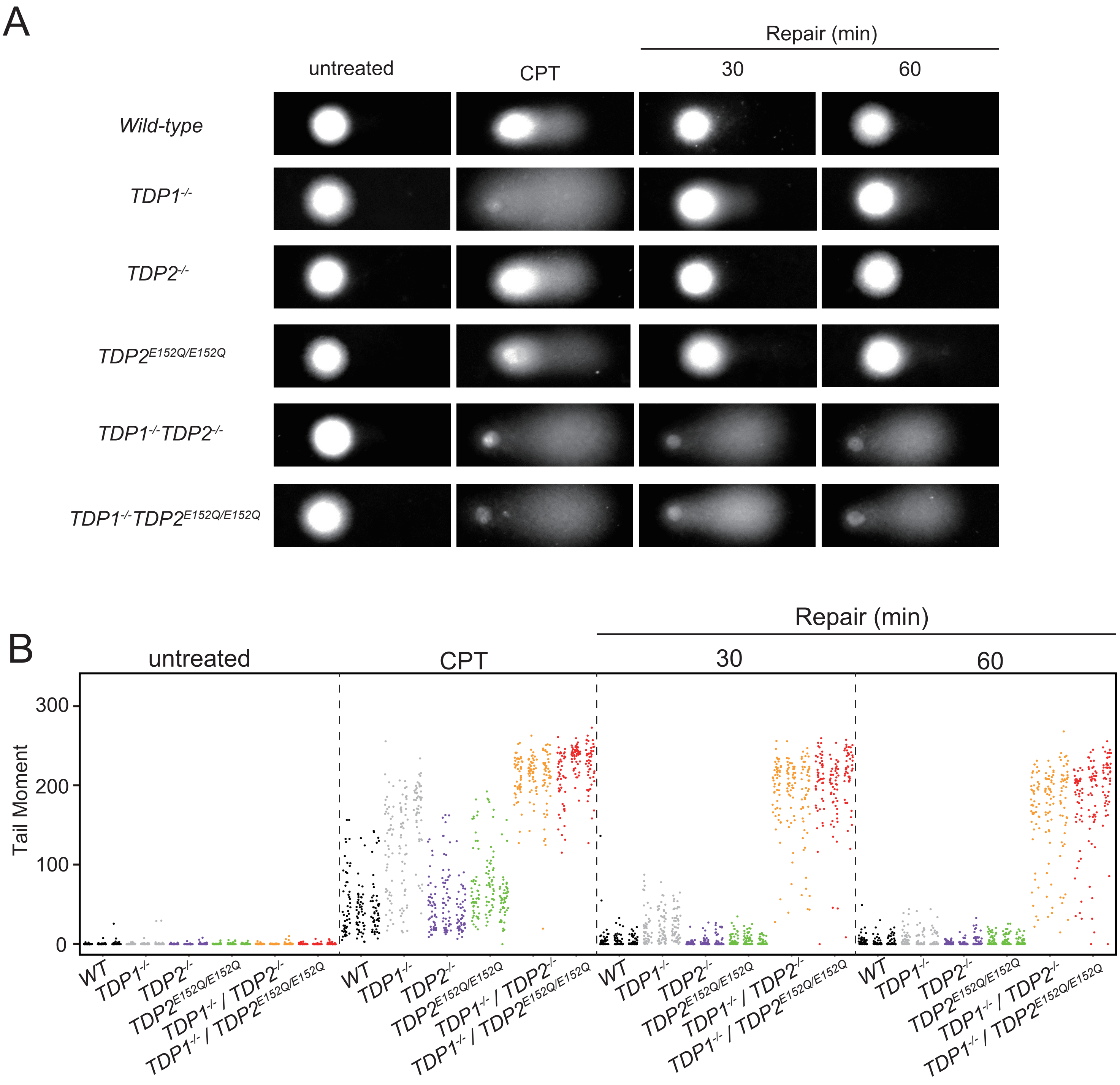

Supplementary Figure S7. Alkaline comet assays used to measure the repair kinetics of SSBs in TK6 mutant cells treated with CPT.

Indicated cells were treated with CPT or left untreated and incubated for 30 and 60 min for repair. SSBs were analyzed using alkaline comet assays (as described in Figure 3D, E). Typical alkaline comet images (A) and the raw data of tail moments before standardization (B) are shown. Data below CPT (A and B) indicate the samples prepared immediately after CPT treatment (0 min repair).

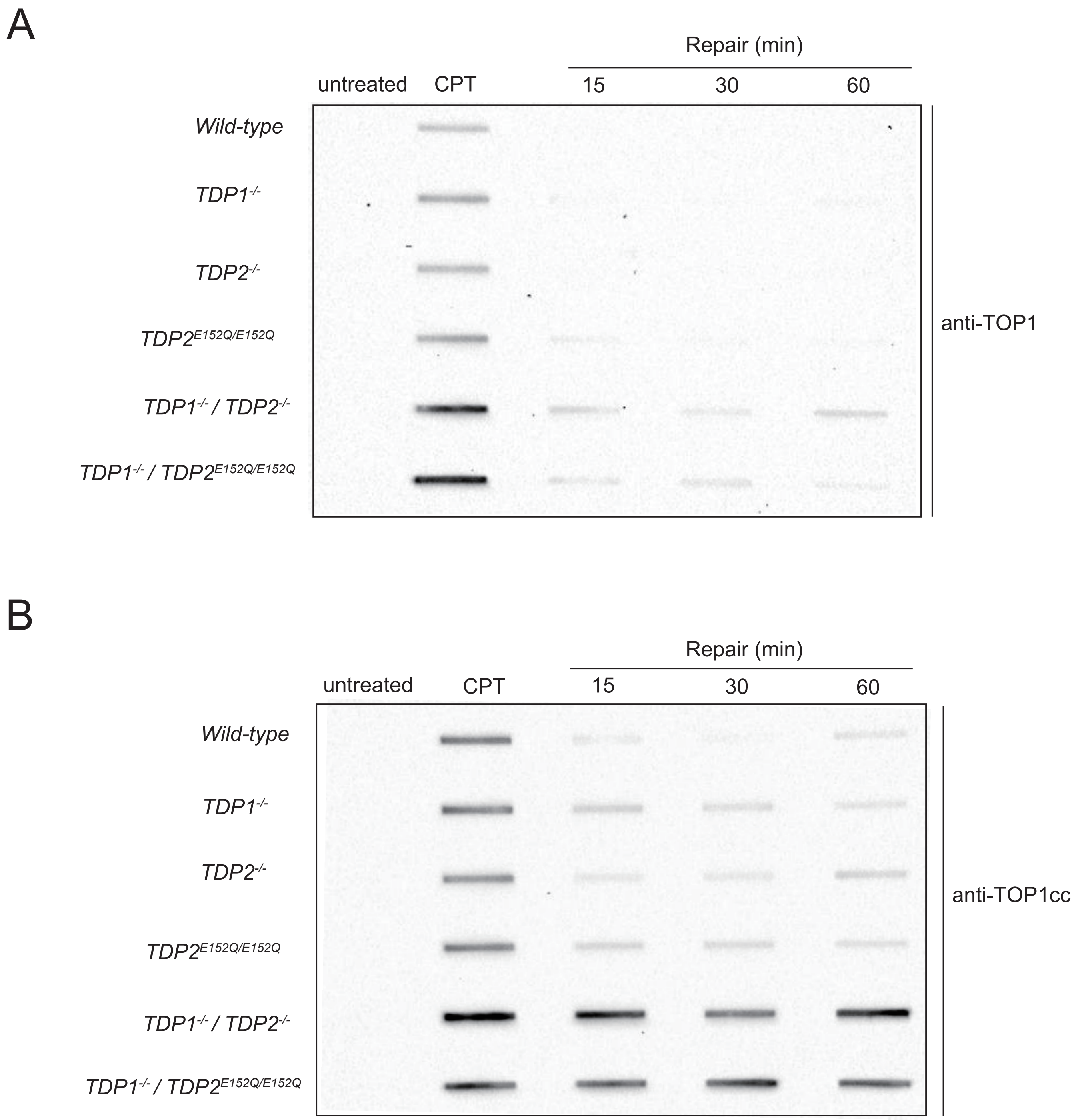

Supplementary Figure S8. Slot-blot analysis used to monitor the first and second steps of the TOP1cc repair process in TK6 mutant cells.

Indicated cells were treated with CPT, and genomic DNA was isolated 15–60 min after CPT treatment and slot-blotted on a membrane (as described in Figure 3F, G). TOP1 and the TOP1-derived peptide that were covalently linked to DNA were detected using an anti-TOP1 antibody (A) and anti-TOP1cc antibody (B), respectively. Typical slot-blot images are shown. The images below CPT indicate the DNA samples prepared immediately after CPT treatment (0 min repair).

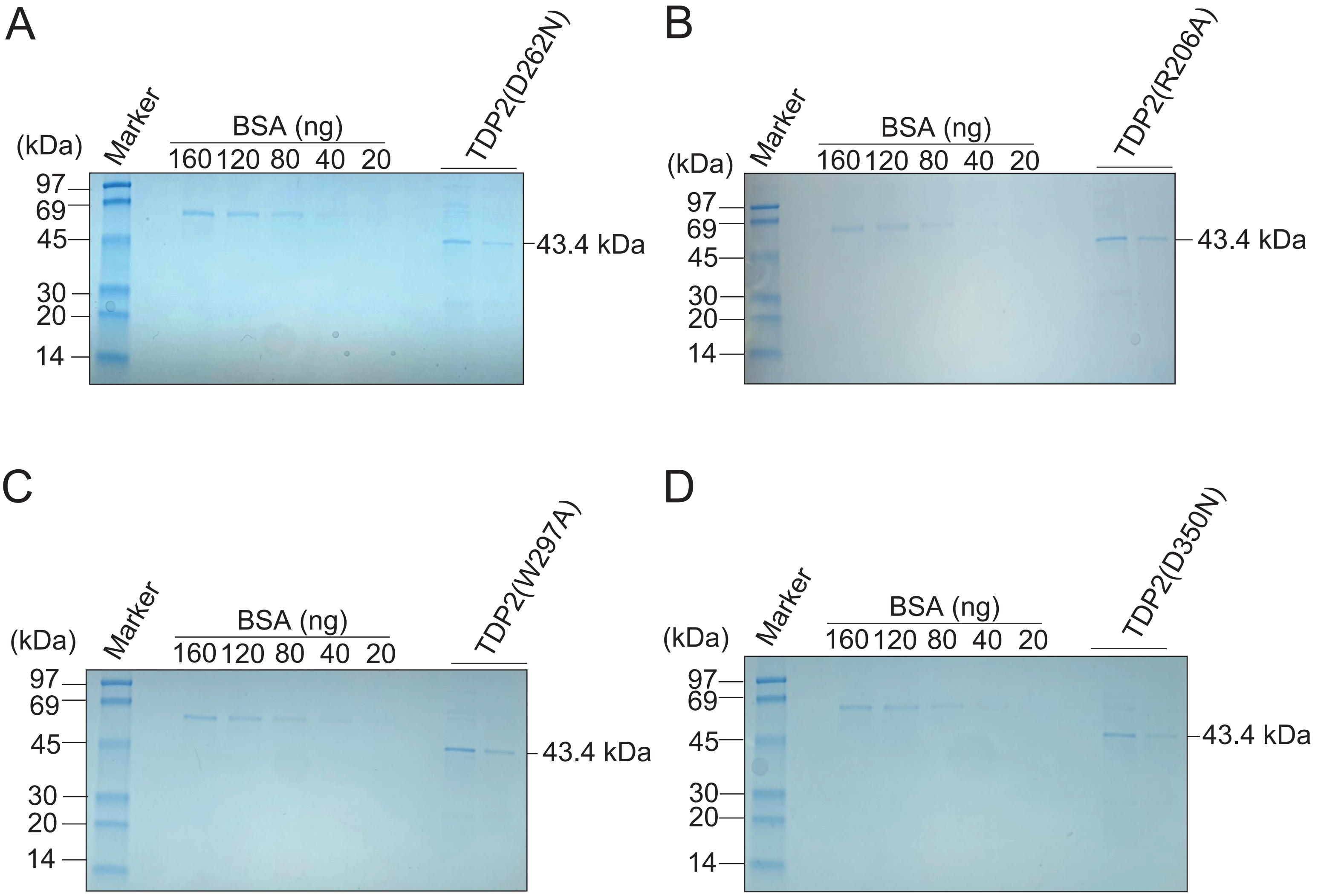

Supplementary Figure S9. SDS-PAGE analysis of the purified mutant proteins of TDP2. Proteins were expressed and purified as described in the Materials and methods. Concentrations and purities of the purified proteins were estimated according to the method provided in Supplementary Figure S2.

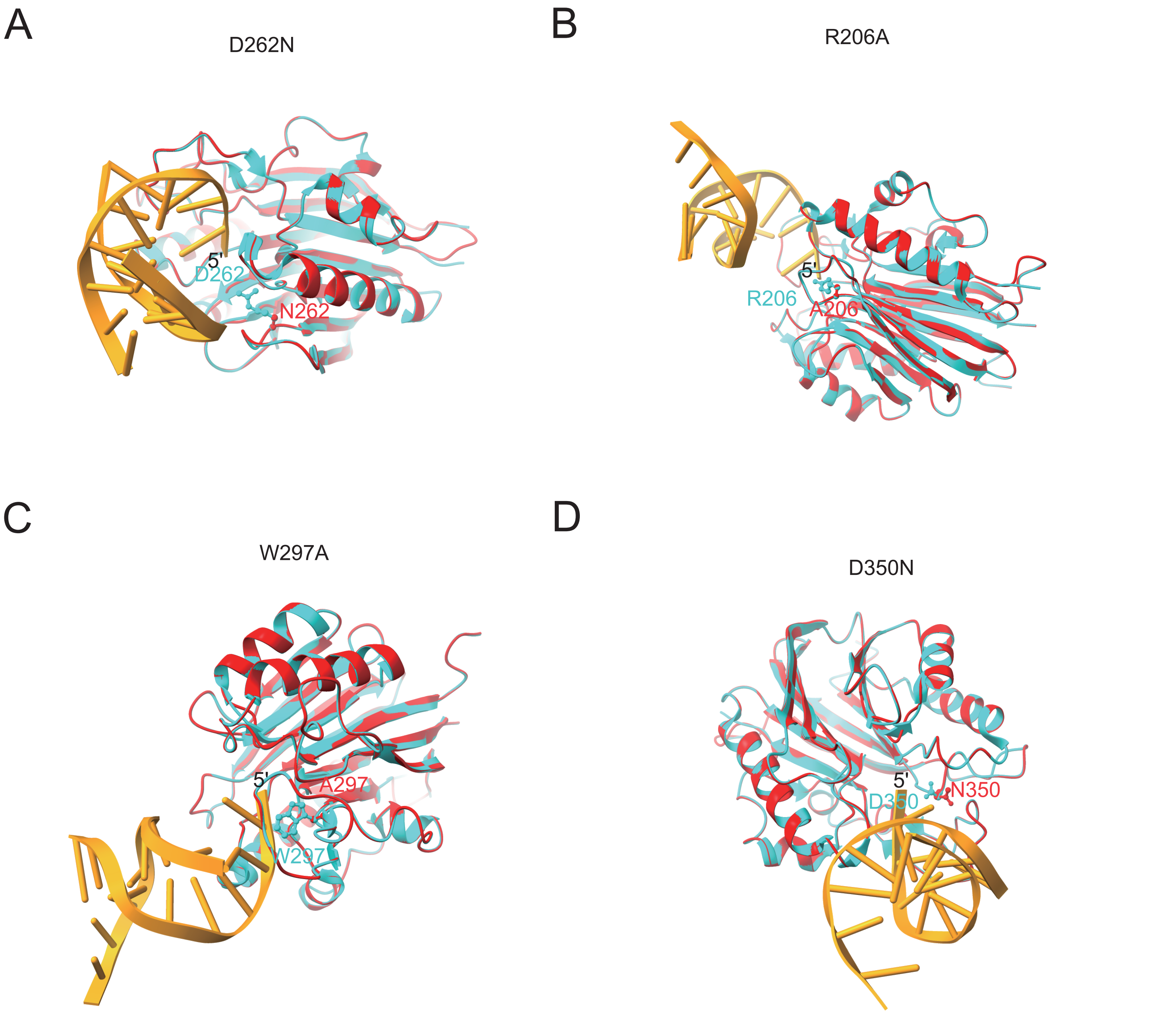

Supplementary Figure S10. Structural comparison of the wild-type and D262N (A), R206A (B), W297A (C), and D350N (D) mutants of human TDP2.

Superpositions of the wild-type (cyan) and mutants obtained by homology modeling (red) are shown. Mutated amino acids in the mutants are shown as ball sticks. DNA is shown in orange with the indicated 5′-end.
